# Avoiding Mitochondrial Apoptosis by the Bcl-2-Driven Bax Oligomerization on Membrane Surfaces

**DOI:** 10.1021/acschembio.5c00913

**Published:** 2026-02-18

**Authors:** Sophie E. Ayscough, Luke A. Clifton, Jörgen Ådén, Sebastian Köhler, Nicolò Paracini, James Doutch, Éilís C. Bragginton, Anna E. Leung, Oliver Bogojevic, Jia-Fei Poon, Tamás Milán Nagy, Hanna P. Wacklin-Knecht, Gerhard Gröbner

**Affiliations:** † ISIS Pulsed Neutron and Muon Source, Science and Technology Facilities Council, 97008 Rutherford Appleton Laboratory, Harwell Science & Innovation Campus, Didcot, Oxfordshire OX11 OQX, U.K.; ‡ 338799 European Spallation Source ERIC, ESS, P.O. Box 176, Lund SE-22100, Sweden; § Department of Chemistry, Division of Physical Chemistry, Lund University, P.O. Box 124, Lund SE-22100, Sweden; ∥ Department of Chemistry, 8075 University of Umeå, Umeå SE-901 87, Sweden; ⊥ Lund Institute for Neutron and X-ray Scattering, Department of Chemistry, Lund University, P.O. Box 124, Lund SE-22100, Sweden; # 56053 Institut Laue Langevin, Grenoble 38042, France; ∇ Electron Bio-Imaging Centre (eBIC), Diamond Light Source Ltd, Diamond House, Harwell Science and Innovation Campus, Didcot OX11 0DE, U.K.

## Abstract

The Bcl-2 family of proteins governs mitochondrial outer membrane (MOM) permeabilization, a critical step in apoptosis that is dysfunctional in many cancers. Although cellular studies have long implicated direct interactions between the pore-forming apoptotic Bax protein and its opponent, the antiapoptotic Bcl-2 protein in apoptosis regulation, the underlying basic principles behind this control remained unresolved. To provide in-depth insight, we carried out a systematic biophysical study in which we utilized neutron reflectometry (NR) and ATR-FTIR to elucidate the molecular communication between those proteins in and around the mitochondrial membrane environment. The spatial and temporal changes across model MOM surfaces were resolved during the interaction of Bax with Bcl-2. The NR-derived membrane surface Bax distributions suggested that Bcl-2 mediated Bax sequestration through both Bcl-2/Bax heterodimerization and Bax/Bax oligomerization. Kinetic analysis revealed a two-step process: rapid formation of Bcl-2/Bax heterodimers, followed by slower Bax oligomerization on these complexes. Importantly, this sequestration mechanism was also observed in the presence of cardiolipin, a lipid known to promote the formation of an apoptotic pore by Bax in the absence of Bcl-2. These findings suggest a fundamental mechanism by which cancer cells may evade apoptosis by exploiting Bcl-2’s ability to neutralize Bax through structural entrapment, even if excess Bax is present, either in response to treatment or natural death signals.

## Graphical abstract

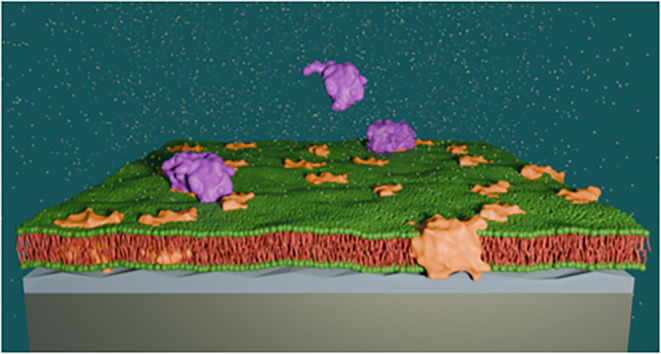

## Introduction

Apoptosis is a form of regulated cell death that is essential for human development and health.^1^ Upon the activation of the intrinsic apoptotic pathway, the progression toward cellular clearance requires the intimate involvement of the cell’s powerhouse, the mitochondria.^2,3^ During this process, the mitochondrial outer membrane (MOM) undergoes permeabilization, releasing apoptotic factors, including cytochrome c. This triggers an irreversible signaling cascade, causing the death of the cell.^3,4^ To avoid undesired clearance of healthy cells, this pathway is critically controlled by the Bcl-2 (B-cell CLL/lymphoma-2) protein family. This family consists predominantly of two main groups of multidomain Bcl-2 proteins called guardians (antiapoptotic/prosurvival) and executioners (proapoptotic/cell killing).^2,3^ To control any apoptotic activity, these opposing family members meet at the MOM, where they interact with each other to determine a cell’s fate: survival by assuring MOM integrity or cell death by inducing membrane leakage.^4,5^

Following apoptotic stimulation, proapoptotic Bcl-2 members such as the prominent Bax (Bcl-2-associated protein X) convert from a monomeric, inactive cytosolic state into a membrane-active one. Upon translocation to the MOM, Bax undergoes further activation steps and dimerization to generate homo-oligomeric structures such as arcs, lines, and ring-like pores in the membrane.^6–10^ All these structures are presumed to perforate the MOM. Recently, atomic resolution insight by Cryo-EM revealed that these previously observed membrane-perforating Bax oligomeric conglomerates are assembled from the same basic Bax (dimer of a dimer) repeating unit,^11^ suggesting that those Bax oligomers function to create pores through the direct rupture of the membrane.

To protect healthy cells from undesired apoptosis by Bax, antiapoptotic guardians such as the membrane-bound Bcl-2 protein preserve mitochondrial integrity through sequestration of activated Bax and its relatives,^2^ a mechanism also exploited by tumor cells to ensure their survival.^12,13^ In nearly 50% of all human cancers, the Bcl-2 protein is involved, often via upregulation, in promoting tumor development and therapy resistance by lowering the cells’ susceptibility to apoptosis, mainly by blocking apoptotic proteins such as Bax.^13–15^ A wide range of *in vivo* and *in vitro* work at the cellular and even tissue level provides clear physiological evidence about the fundamental role of this direct Bcl-2 complexation/inhibiting of Bax.^2,14,15^ Indeed, it has been noted using *in vivo* cell systems that Bax and Bcl-2 can interact directly with very high affinity to prevent the initial stages of apoptosis.^12,13,16–18^ Co-localized Bcl-2/Bax complexes are even visible in tumor tissues using combined Bcl-2/Bax histopathological staining.^19,20^ Using cell lines suppressing antiapoptotic Bcl-2 protein’s expression enabled expressed Bax to directly translocate to the MOM and trigger apoptosis, clearly showing the essential role of Bcl-2 proteins to control Bax activities at the MOM level.^21^

In the last decades, immense progress has been made in unravelling the cellular and molecular mechanisms of apoptotic regulation by the Bcl-2 family; most recently, in providing atomic resolution insight into Bax oligomers causing MOM pore formation and cytochrome c release.^11^ However, the underlying machinery by which the antiapoptotic Bcl-2 protein at the mitochondrial outer membrane level sequesters Bax into a tight complex to prevent its deadly action is still not understood at the fundamental level; mainly due to the lack of in-depth molecular insight into the generation and organization of those apoptosis-preventing protein assemblies.^2,5^

The structure of cytosolic Bax^22^ and the oligomeric repeating subunit of activated Bax exist.^11^ However, no structure has been resolved for the hydrophobic, membrane-located full-length human Bcl-2 (239 aa). Structures have been resolved for truncated chimeric Bcl-2 variants (often 166 aa^23^), which are not membrane active nor functional *in vivo*. Therefore, for intact human Bcl-2, mainly due to its difficulties in being produced in sufficient amounts and being insoluble, only a few basic molecular studies exist, mainly using cell assays, truncated Bcl-2 versions, or comparisons with its soluble relative Bcl-X_L_.^12,13,16–18,24–30^ Only recently has an intact and fully functional human Bcl-2 protein become available in mg amounts,^31^ which enabled us to determine its location as a fully inserted protein in its target membrane under apoptotic stress conditions.^32,33^

Here, we resolved the molecular-level details of the cell-protecting function of Bcl-2. Neutron reflectometry (NR) and attenuated total reflection Fourier transform infrared spectroscopy (ATR-FTIR)^9^ have allowed us to follow the spatial and temporal fate of the Bcl-2 and Bax proteins during their interplay at the membrane level. NR with sample and solution hydrogen isotope labeling allows for the location and changes in the distributions of the lipids and proteins across model membranes to be resolved quantitatively with molecular-level resolution across the surface (perpendicular to the interface).^34,35^ This technique was used to structurally probe the interactions of Bax with lipid bilayer models of the MOM to examine how the presence of Bcl-2 in the bilayer modifies Bax’s pore-forming activity.^9,32^ ATR-FTIR was used to examine the membrane-association kinetics of Bax in the presence of Bcl-2. It is a surface-sensitive spectroscopic technique that allows for the changes in IR adsorption bands around the solid–liquid interface to be monitored. Here, ATR-FTIR was used to monitor the relative changes in the protein and lipid contents of the membrane mimics against time via the specific IR absorption bands of these components.^9,35^ Finally, electron microscopy (EM) imaging of Bcl-2-containing vesicles was used to complement the NR data by directly imaging the changes in membrane morphology before and after the interaction with Bax. Taken together, we observed that Bcl-2 not only sequesters Bax in direct complexes at the membrane level but, surprisingly, also induces Bax oligomers to arrange into extended assemblies of repeating subunits.^11^ These findings provide molecular details of the fundamental process of apoptosis regulation. This information is essential to understand the upstream physiological consequences of Bcl-2 overexpression in many tumors.^12,13,16–18,24–28^

## Results

Neutron reflectometry was used to provide quantitative structural insights into the molecular machinery by which Bcl-2 inhibits Bax and prevents apoptotic pore formation at the membrane level. For these studies, a series of MOM membrane mimicking models, composed of phospholipids and Bcl-2, was deposited on silicon substrate surfaces via vesicle fusion.

Model biomembranes reflect key features of *in vivo* systems. When combined with physical and structural analysis techniques,^36^ these models provide a means of resolving the molecular-level details of membrane biochemical processes, which are not currently resolvable in live organisms.

The MOM models were supported lipid bilayers (SLBs) composed of either POPC (1-palmitoyl-2-oleoyl-*sn*-glycero-phosphocholine) only or POPC and 10% (mol/mol) of the anionic phospholipid tetra-oleoyl cardiolipin (CL), with varying amounts of embedded Bcl-2. These simplified POPC and POPC/CL lipid compositions have been shown by us and others to mimic the basic physicochemical features of the MOM. Indeed, studies using bilayers of increasing complexity, including mitochondrial lipid extracts and intact mitochondria, showed the same basic behavior in response to apoptotic stimuli,^10,37–41^ as found for simplified membrane models composed of phosphatidylcholine/cardiolipin lipid mixtures.^9,40,42–44^

Multiple MOM models were examined to probe how the presence of Bcl-2 changes the interaction of Bax with the MOM. These contained different amounts of the antiapoptotic Bcl-2,^23^ and, in some cases, the proapoptotic and mitochondrial-specific lipid, cardiolipin.^45^

### Bcl-2 Sequesters Bax on the Membrane Surface, Preventing Pore Formation

Prior to the titration of Bax, Bcl-2-containing POPC vesicles were used to form SLBs at the silicon/water interface. These were then characterized by NR, a technique that resolves the structural distribution of macromolecular components across interfaces. To obtain a volume fraction profile of the membrane, NR data sets measured in solutions of different H_2_O/D_2_O buffer ratios were co-refined, as seen in the NR data and the corresponding data analysis in Figure 1. The scattering length density and component volume fraction profiles resulting from the best model fits to the NR curves (Figure 1C,D) showed that Bcl-2 was located within the lipid bilayer. This membrane inclusion of Bcl-2 is in agreement with previous observations on similar model membrane systems,^32,46^ and even as seen in cellular membranes upon apoptotic stimuli.^29^ Electron microscopy imaging of protein–lipid vesicles supported this analysis, showing the presence of immersed Bcl-2 within the vesicles (Figure 1F).

**Figure 1:**
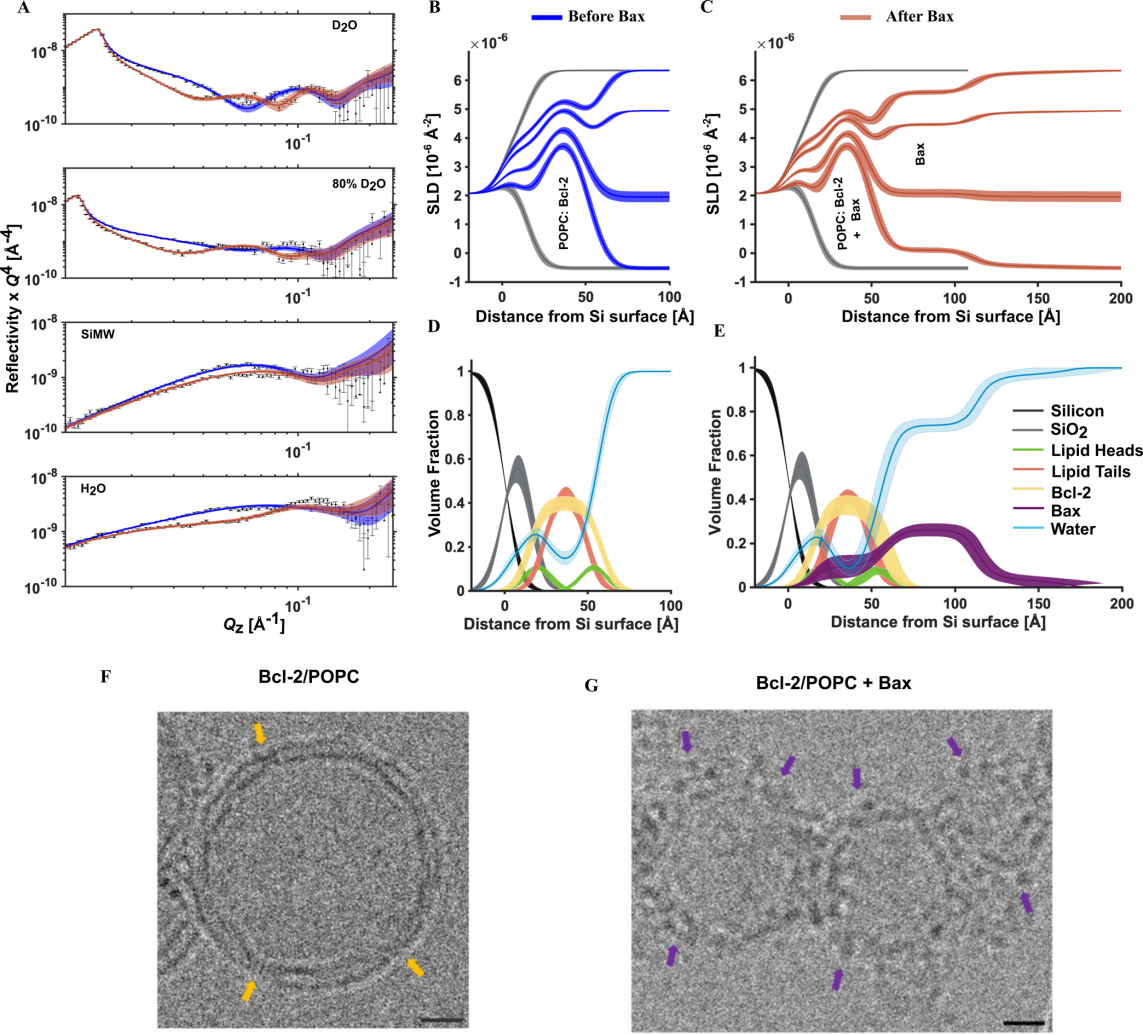
Bax targets Bcl-2-containing membranes without poration. NR data (A, error bars) and model-data fits (A, lines; X^2^ = 34.5, see also Supporting Information Table 1) from a d-POPC/Bcl-2 SLB before (blue) and after (red) the presence of natural abundance hydrogen (h-)­Bax are shown in four differing solution isotopic contrast conditions. The corresponding scattering length density (SLD) profiles are shown for the surface structure before (B) and after the h-Bax interaction (C), together with the corresponding component volume fraction profiles (D, E). Individual components are color-coded as indicated, with the Bcl-2 distribution colored orange and the Bax protein distribution in purple. Complementary Cryo-EM images of Bcl-2/POPC vesicles clearly indicate the presence of Bcl-2 (yellow arrows) within the lipid bilayer (F) and the binding of discrete Bax distributions (purple arrows) onto the vesicular surface without disruption (G), consistent with the NR findings. The scale bar is 10 nm.

The interaction of Bax with the POPC/Bcl-2 SLB (see Figure 1) led to the distribution of Bax predominantly on the surface of the membrane. In repeated measurements (see Figures 3, S2, and S3), a minor bilayer thickening (∼2 Å, see Table S4) was observed, likely due to partial Bax penetration into the SLB. In the absence of Bcl-2, Bax’s interaction with the POPC SLB led to membrane disruption by pore formation and the transfer of lipids into protein–lipid complexes on the bilayer surface, as seen in Supporting Information Figure S1 and Table S1. A mechanism elucidated by us previously.^9^

This mechanism was not observed with POPC/Bcl-2 films (Figures 1 and 3). Combined, these results revealed that the presence of the antiapoptotic Bcl-2 changed the nature of the Bax interaction with the MOM mimics, preventing membrane perforation or disruption.

The amount of Bax on the membrane surface showed a direct correlation with the Bcl-2 content of the MOM models (Figure 3 and Table 1). This indicated a direct interaction between the two proteins, likely the formation of Bcl-2/Bax complexes at the membrane interface, consistent with previous observations.^46,47^ The observed distribution of Bax away from the membrane surface (Figures 1C and 3A–C) suggests that Bcl-2 not only sequestrated Bax into a 1:1 complex but also induced Bax oligomers to form on the outer bilayer surfaces (see Figure 1E).

**Table 1:** Comparison of Bound Bax to Bcl-2 Content in the MOM Models Studied by NR, where No Membrane Disruption was Observed^a^

**lipid composition**	**POPC**			9:1(mol/mol) **POPC:CL**	
**Bcl-2 volume coverage in bilayer/%**	9.9 (5.2–14.4)	14.2 (9.3–19.2)	39.9 (36.6–43.3)	24.7 (23.0–26.4)	38.7 (36.8–40.5)
**Bax surface bilayer proximal distribution thickness/Å**	88.4 (64.9–116.3)	67.8 (56.5–85.7)	55.0 (50.6–56.8)	54.6 (50.1–58.8)	64.7 (61.4–68.2)
**Bax surface bilayer proximal distribution coverage/%**	13.0 (10.3–15.8)	8.0 (5.0–11.2)	26.2 (23.2–29.1)	19.8 (16.8–23.5)	29.3 (26.5–33.2)
**Bax surface bilayer distal distribution thickness/Å**	80.8 (58.6–94.3)	n/a	52.7 (24.5–76.9)	n/a	n/a
**Bax surface bilayer distal distribution coverage/%**	5.8 (3.7–7.9)	n/a	4.5 (2.4–9.2)	n/a	n/a
**total membrane-bound Bax layer thickness/Å**	163 (142–192)	68 (56–86)	108 (74–134)	55 (50–59)	65 (61–68)

a MOM-bound Bax layers of similar coverage within the total membrane surface protein envelope were assigned as the distinct Bax distributions described here.

To provide molecular insight into the membrane surface Bax oligomeric structures, we compared Bax’s basic structural features^8,11,22^ (see Supporting Information Figures S13, S14, Tables S6, and S7) with the membrane surface protein distributions found by NR. The length scales of the steady-state Bax distributions found in the presence of Bcl-2 (see Figures 1, 3, and Table 1) suggested that Bax formed distributions corresponding to ∼2–4 vertical protein units on the bilayer surfaces (see Figure 4). These tapering distributions of oligomerized Bax were most clearly seen bound to the surface of the ∼40% Bcl-2 containing the POPC/Bcl-2 SLB (Figure 1). Here, a 20% coverage of Bax was found in a ∼55 Å membrane proximal distribution, which decreased sharply to 6% for an additional ∼53 Å Bax distribution bound to this. The ∼55 Å plus ∼53 Å tapering distribution is suggestive of a high-coverage layer of Bcl-2-bound Bax oligomers (possibly dimers or tetramers; see Supporting Information Tables S5 and S6) on the outer surfaces of bilayers to which additional Bax oligomers were bound, forming larger multimeric assemblies. Consequently, the Bax assemblies cannot access the membrane to generate pores.

EM imaging of the binding of Bax to Bcl-2-containing POPC vesicles supported the NR-derived findings. Prior to Bax binding, Bcl-2 was observed to be embedded in the lipid bilayer as distinct “notches” (see Figures 1F, S10, and S11), in agreement with previous observations on similar biosystems.^48^ Upon the binding of Bax to the vesicle surface, no disruption of the vesicles^47^ was observed, and discrete distributions of the Bax proteins on the membrane surface were observed, similar to the Bcl-2-bound Bax layer revealed by NR (see Figures 1G, S10, and S12). High-resolution structures of Bax assemblies by Cryo-EM^11^ support this finding.

### Kinetics of the Bax/Bcl-2 Complex Formation

A combination of time-resolved (TR-) NR and ATR-FTIR was used to examine the time-dependence of the Bax sequestration process by Bcl-2. TR-NR provided time-dependent structural details on the changes in the macromolecular distributions in and above the Bcl-2-containing MOM models during the interaction of Bax, while ATR-FTIR complemented this by revealing the relative changes in macromolecular components of the MOM models via their IR absorption bands (notably the change in the protein amide I band).

These measurements were undertaken to examine how, upon initial molecular complex formation, Bax can oligomerize into large, nonperforating structures at the Bcl-2 loci, as visible in the steady-state NR results in Figure 1 and Cryo-EM pictures (Figure 1G). An ATR-FTIR analysis of Bax’s interaction with a Bcl-2-containing deuterated (d-)­POPC bilayer revealed a two-stage binding process via the time-dependent increase in the protein amide I band. This consisted of an initial fast component with a time constant of 9 ± 1 min and a slower secondary process with a time constant of 148 ± 11 min (Figure 2C,D). TR-NR analysis had a lower time resolution (∼15 min for TR-NR vs 80 s for ATR-FTIR). However, the analysis of the change in membrane surface protein content uncovered the slower secondary process found in the ATR-FTIR measurements (time constant of 163 ± 11 min, Figure 2D, inset).

**Figure 2:**
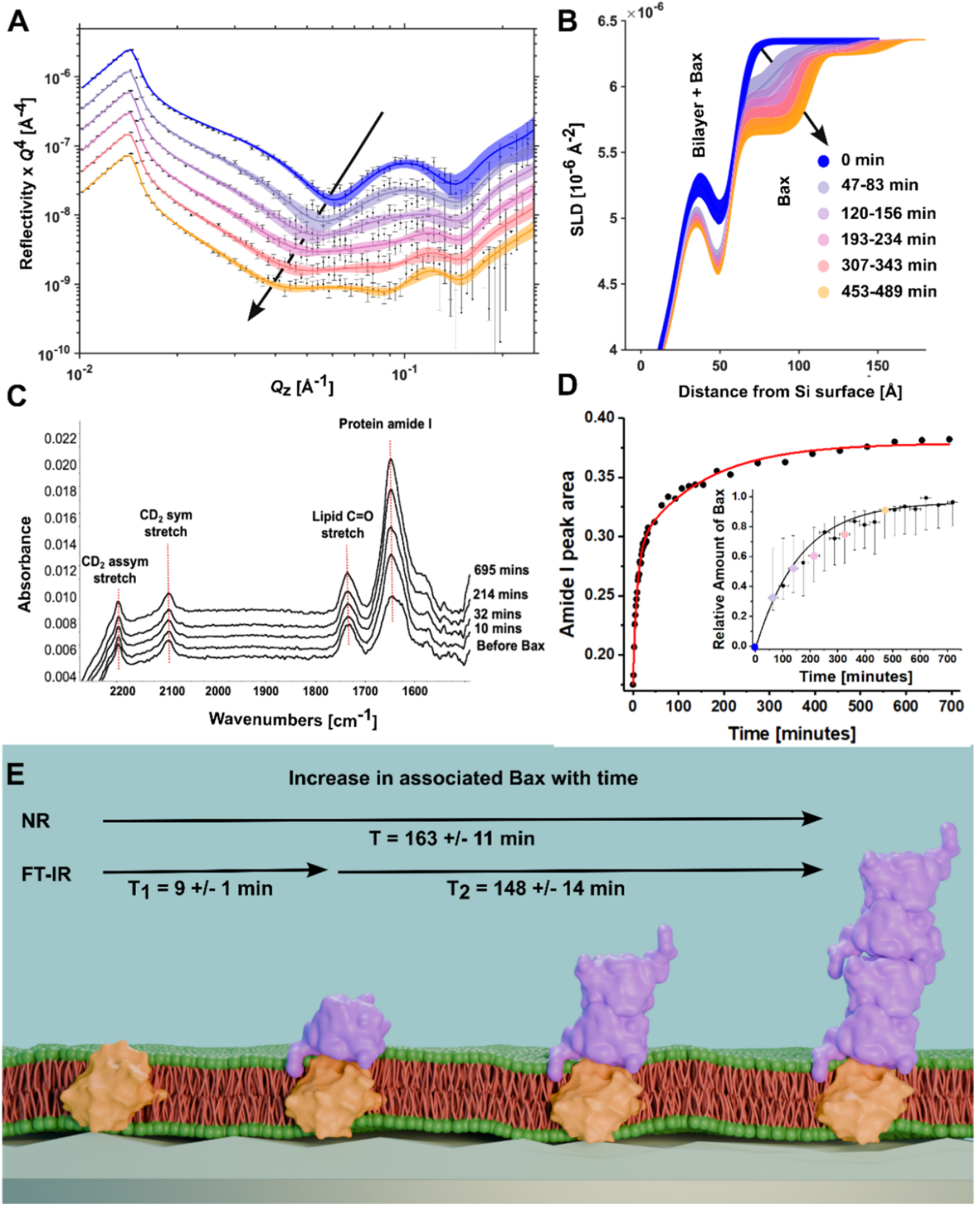
Kinetics of Bax sequestration by Bcl-2 at membrane level: from initial contact to oligomerization: Time-dependent TR-NR data (error bars) and model-data fits (lines, X^2^ = 52.0) in the D_2_O solution contrast obtained during Bax binding to Bcl-2-containing d-POPC bilayer are shown in (A) and as time-dependent SLD profiles in (B) showing the increased accumulation of Bax on the bilayer surface; black arrows indicate the change with time. Complementary ATR-FTIR data in (C) depict the increase in amide I during Bax binding with time. The corresponding analysis (D) revealed a two-stage kinetic process with a fast (9 min) progression, followed by a slower (148 min) progression (see also Supporting Information Figures S8 and S9). Inset in (D) presents the kinetic analysis of the NR data showing the increase in Bax volume fraction with time (see the Supporting Information and Table S5 for details). Each colored point represents a different NR data set and matches the data sets shown in (A) and (B). An analysis of the increase in Bax protein on the membrane surface reveals a process ranging from initial binding of Bax monomers to oligomerization on this surface. A schematic representation of this process is shown in (E), with protein crystal structures of Bax (1F16 ^22^) and Bcl-2 (1G5M^23^) representing the distribution of these proteins across the POPC/Bcl-2 SLB and how this changes with time. It should be noted that we do not expect Bax to be in its solution folded state in the Bcl-2-bound clusters.

TR-NR analysis revealed the temporal evolution of Bax oligomers on the POPC/Bcl-2 surface. Initially, a predominantly monomeric layer of Bax (∼25 Å thickness, see Supporting Information Tables S5–S7) was bound from solution onto the SLB surface; a fast event most likely reflected in the fast process revealed by ATR-FTIR. Following this, the thickness of the Bax layer doubles to a ∼50 Å layer, consistent with a Bax oligomer formation, which increases in the membrane surface coverage. Finally, an additional Bax oligomer layer of resolvable coverage (>3%) appears to be bound to this, which also increases in surface coverage (see Figure 2B and a schematic interpretation of this in Figure 2E). The oligomerization of Bax on the membrane surface is interpreted to be a slow secondary binding process, as revealed by both ATR-FTIR and TR-NR.

### Cardiolipin Enhances the Membrane Surface Anchoring of Bax Oligomers by Bcl-2

The mitochondria-specific lipid CL plays a key role during apoptosis in facilitating the recruitment of Bax to the MOM and its perforation.^9,38,39,49,50^ The presence of CL accelerates and amplifies Bax’s perforation activity in the absence of Bcl-2 in the membrane,^51^ compared to the behavior seen for CL-free POPC bilayers (Figure S1). Based on lipid analysis,^52,53^ there is up to 4% CL on average in the MOM, which increases to 20% in areas close to membrane contact sites and junctions. Therefore, here we used 10% CL in Bcl-2-containing POPC/CL SLBs to study the inhibitory effect of Bcl-2 on Bax in the presence of the poration-promoting CL.

Bcl-2 in CL-containing SLBs sequestered Bax in a similar way as was observed for POPC-only bilayers (see Figure 3). Only with both the proapoptotic CL (10% mol/mol lipid) and a relatively low amount of the antiapoptotic Bcl-2 present (6% volume fraction) was the competition between the Bcl-2 and CL for Bax observed, leading to a mixture of Bax/Bcl-2 complexes and Bax/lipid clusters (following poration) on the bilayer surface (see Figure 3F and Table 1). Larger Bcl-2 volume fractions within the SLBs led to Bax oligomers only on the POPC/CL/Bcl-2 SLB surfaces (Figure 3 G,H). As with the POPC/Bcl-2 SLBs, there was a direct correlation between the Bcl-2 content and the volume fraction of Bax oligomers on the bilayer surfaces (Figure 3G,H and Table 1). Indeed, a ∼25% Bcl-2 content produced a ∼20% bound Bax coverage and a ∼40% Bcl-2 content caused ∼30% Bax coverage on the membrane surface. These Bax layers had distributions more closely associated with the SLB surface than the equivalent Bax oligomers found on the CL-free POPC/Bcl-2 surfaces, being 55 and 65 Å across two independent measurements (see Table 1), which is suggestive of Bax oligomers (see Supporting Information Section 3). This observation, combined with the lack of a membrane-embedded Bax distribution, suggests that CL plays a role in Bcl-2/Bax complex formation, possibly forcing Bax oligomerization along the membrane surface rather than away from its surface into solution, as was observed for the Bcl-2/POPC systems.

**Figure 3:**
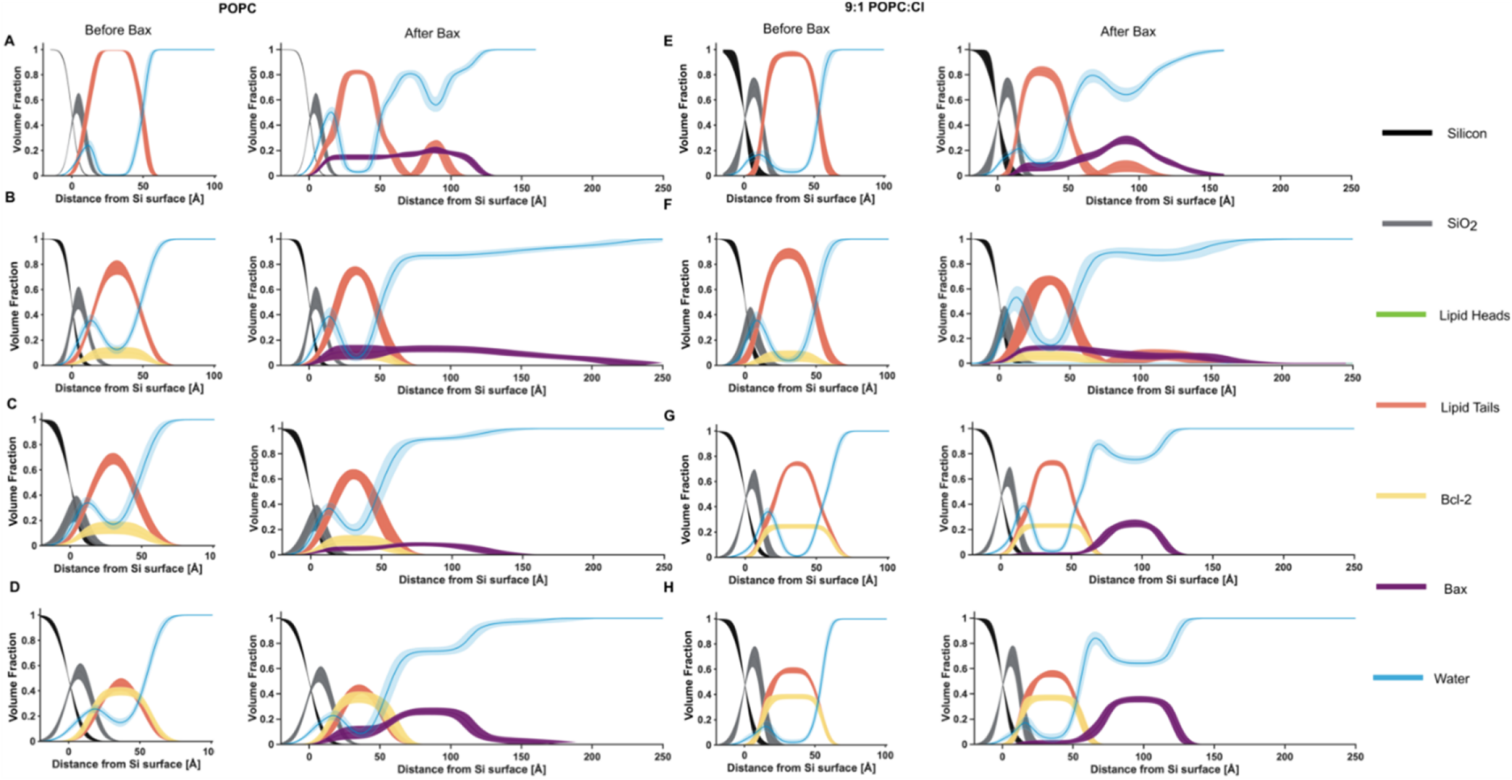
Positive correlation between bound Bax and the SLB Bcl-2 content. Interaction of Bax with lipid bilayers containing an increasing volume fraction of Bcl-2, in POPC bilayer (A–D) and 9:1 POPC:CL bilayer (E–H) SLBs. Component volume fraction profiles determined from NR data analysis before (left) and after (right) the interaction of Bax with the MOM models are shown. Individual components are color-coded as indicated in Figure 1, with the Bcl-2 distribution in orange and the Bax protein distribution in purple. NR data sets, model-data fits, and SLD profiles used to determine these volume fraction profiles are shown in Figures 1 and S1–S6. The data and model for E are taken from Clifton et al.^9^

## Discussion

This study used a range of structural and biophysical techniques to resolve the influence of the antiapoptotic Bcl-2 on the interaction of the proapoptotic Bax with models of the MOM. The analytical techniques used (NR, EM, and ATR-FTIR) were optimal for resolving the changes in the distributions of macromolecular components in and around the complex model biological membranes under study. Analysis revealed that the presence of Bcl-2 in the MOM models prevented Bax-induced membrane poration (which was found to occur in the Bcl-2-deficient cases). The binding of Bax protein to the bilayer surface is a two-stage process: initially binding as a monomer, which is then oligomerized over time. The amount of Bax found to bind to the Bcl-2-containing MOM models was directly proportional to the Bcl-2 content, suggesting Bcl-2/Bax complex formation. However, the resolution of the measurements was at the molecular level and thus was neither suitably high to provide atomistic level insights (i.e., protein residue specific) into the nature of Bax/Bcl-2 complexation nor was it clear why this induced Bax to aggregate on the membrane surfaces. Therefore, further studies are needed to fully resolve the Bcl-2-induced Bax aggregation described here. Below, we interpret our findings in light of the large amount of current literature on the Bcl-2 protein family.

The observed sequestration of Bax by membrane-embedded Bcl-2 described here may reflect the key features of the proposed Bcl-2 action *in vivo*, such as in Bcl-2 overexpressing cancer cells, namely, the inhibition of Bax from initiating apoptotic cell death.^2,15,25,54^

Heterodimerization of Bcl-2 and Bax has long been hypothesized as the key apoptotic-blocking mechanism, which has been largely investigated through coprecipitation, protein binding assays, and cell-based studies.^25,55^ The Bcl-2 protein contains a hydrophobic protein core, with its BH3–BH1–BH2 region forming an extended groove interface, which recognizes apoptotic proteins like Bax via their specific Bcl-2 homology 3 (BH3) death motifs,^23,25,56^ a process also seen for Bcl-2’s close soluble relative Bcl-x_L_.^27^ Those initial complexes are structurally quite similar to Bcl-2 (PDB: 2XA0)^25^ and Bcl-x_L_ (PDB: 3BL7).^27^ After this initial BH3 domain-binding step, further domains of the Bax engage with the Bcl-2 to generate a Bcl-2/Bax complex with further increased affinity in the low nM region,^56^ as seen *in vitro* ^46,57^ and *in vivo* studies.^58^

In the time-dependent NR experiments described here (Figure 2), Bcl-2/Bax complexation occurred in two kinetically visible stages. Initially, the formation of Bcl-2/Bax heterodimers across the model membrane was observed, which happened via a fast (∼9 min) initial Bax association, which is presumably triggered by initial Bax-BH3 motif binding to the Bcl-2 groove and then followed by the formation of a Bcl-2/Bax 1:1 complex, as seen in Figure 2B. The Bax monomer described by TR-NR analysis here was likely in a preactivated state, since the nonactive Bax monomer is known to have a longer lag time for membrane poration compared to a detergent-activated form.^59^ Previous studies have shown that the time scales for Bax–membrane interaction range from minutes to hours.^10,11,39,42,43,50^ In most cases, Bax becomes activated during purification due to contact with lipids/membranes in a similar manner to surfactants^22^ or tBid.^38^

Results presented here suggest that initially, Bcl-2 proteins sequester membrane-associated Bax into a 1:1 complex (Table S5 and Figure 2), presumably via its exposed BH3 motif, as established previously.^2,25,54^ This sequestration interaction occurs at the membrane level, as depicted in Figure 2E, and presumably prevents the onset of pore formation. The Bcl-2/Bax complex formation was also observed in physiological studies on apoptosis.^12,13,16–18,58^ In tumor tissue, the Bcl-2 inhibition of Bax into protein complexes was resolved using combined Bcl-2/Bax histopathological staining,^19,20^ and mutagenesis studies on Bcl-2 and/or Bax further supported these findings.^11,25,56,58,60^

We observed a slower second step in the kinetics of binding of Bax to the Bcl-2-containing SLBs. NR characterization of the final steady-state structures showed a Bax distribution consistent with Bax oligomerization onto and away from the Bcl-2-containing SLB surface, as illustrated in Figure 4. Bax–Bax oligomerization has been characterized previously as active and inactive cytosolic Bax dimers^22,61–63^ and is further observed in its pore formation in the absence of Bcl-2.^8,11,43,64,65^ As recently resolved by Zhang et al.,^11^ Bax forms oligomeric structures from repeating subunits of Bax tetramers with Bax being able to extend its polymerization via its α9 helices. Extrapolating this observation to the results presented here would suggest that, starting from a membrane-bound Bcl-2/Bax 1:1 assembly, Bax can expose parts of its core domain (α1–α5 helices), acting as an initial site for further Bax–Bax polymerization. Thereby, oligomerizing Bax into regular structures away from the membrane prevents pore formation (Figures 2 and 4). Indeed, despite the vast excess of Bax compared to Bcl-2 in the studies presented here, lipid removal, and therefore pore formation, was only observed with a low Bcl-2 content in the membrane, as seen in Figure 3F. Here, Bcl-2 abundance was so low that it could only inhibit a smaller population of Bax, while the major population of Bax could still perforate by removing lipids and depositing them on the membrane surface; a situation clearly visible in the broad Bax distribution in the plot and the lipid population above the membrane (in red), typical for membrane perforation.^9^ However, in the presence of sufficient Bcl-2, no Bax-induced membrane damage could be observed (Figure 3, except for A, E, and F) despite an excess of Bax during titration in the aqueous phase. Therefore, it could be speculated that this Bcl-2-induced Bax oligomerization may play a role not only in tumors with high Bcl-2 overexpression levels^13,19,20^ but also in tumors with slightly increased Bcl-2 levels.^13–15^

**Figure 4:**
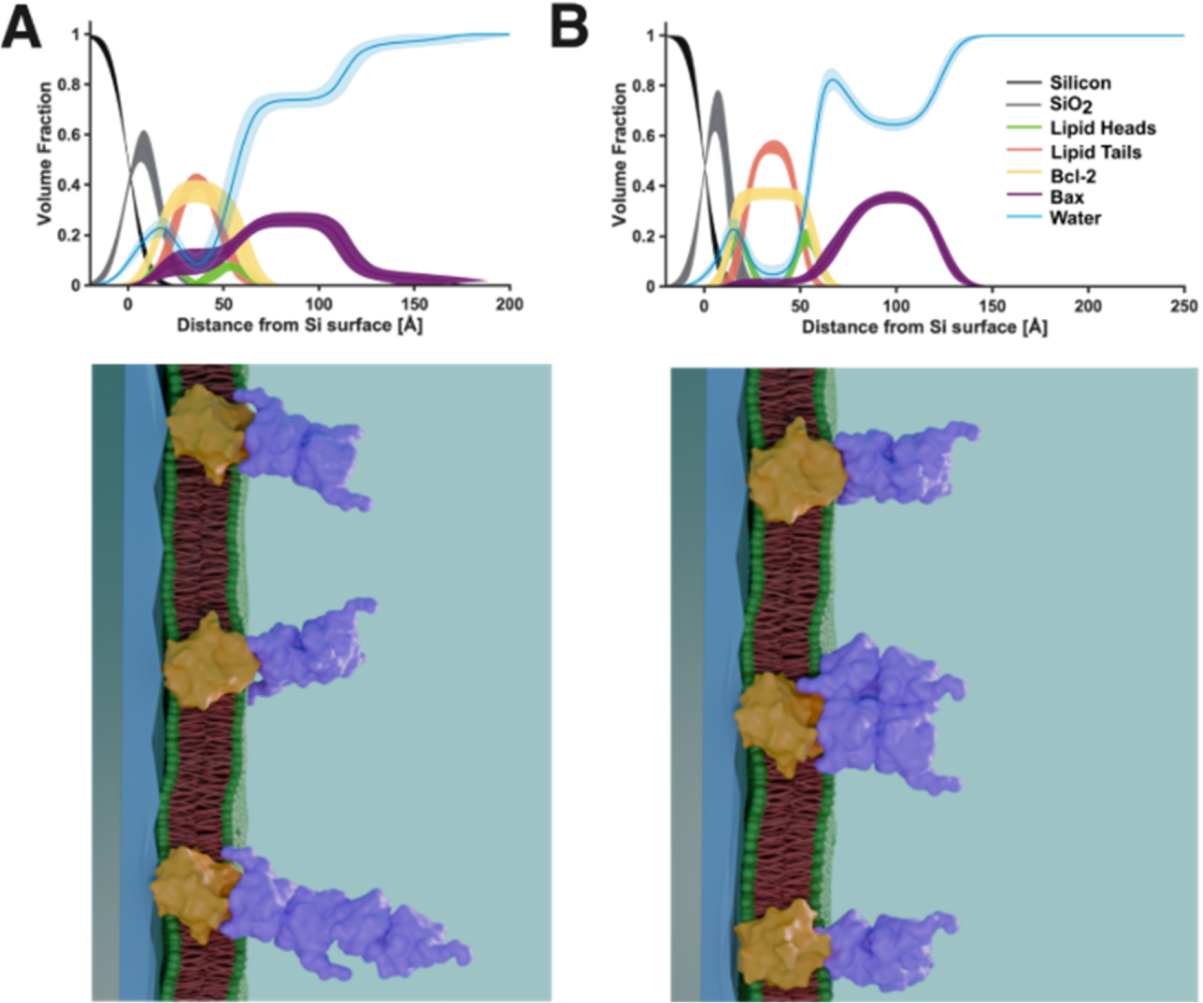
Comparison of NR-derived component volume fraction distributions from Bax binding to SLBs containing Bcl-2 and schematic representations of the derived distributions of components across these protein–lipid membranes. Bax binding to Bcl-2-containing SLBs composed of POPC (A) and POPC/CL (B) was associated with Bax–membrane surface binding in density profiles, which suggested Bax oligomerization. The distribution of the differing molecular components across the SLB surfaces is depicted with the protein crystal structures of Bax (1F16 ^22^) and Bcl-2 (IG5M^23^). It should be noted that we do not expect Bax to be in its solution folded state in the Bcl-2-bound clusters.

It could also be speculated that Bcl-2 overexpressing cancer cells are able to resist apoptosis initiation in the presence of excess activated Bax via Bcl-2-mediated oligomerization of Bax away from the membrane to avoid pore formation. It could be postulated that healthy cells could retranslocate transiently Bcl-2-bound Bax into the cytosol and convert them into their nonactive soluble state via a mechanism involving Bcl-x_L_ and further members of the Bcl-2 family.^66,67^ In cancer cells, this mechanism might be coupled with removing excess Bax via protein degradation or other processes. Nevertheless, Bcl-x_L_, whose tail-anchored structure in nanodiscs is known^24^ (in contrast to intact Bcl-2), also becomes membrane active in the presence of Bax in a similar way by undergoing major structural membrane-associated arrangements. This is typical for tail-anchored membrane proteins; as pointed out by Hill et al.^30^ However, Bcl-x_L_ still has fundamental differences at the membrane with respect to Bcl-2, e.g., by engaging with other BH3-only, apoptosis-regulating proteins.^2,39,40,67^

Nevertheless, the lipid composition of the MOM plays a role in intrinsic apoptosis. The presence of the mitochondria-specific anionic lipid CL significantly increases the membrane perforation activity of Bax in the absence of Bcl-2.^9,37,45,50^ However, as described here, if Bcl-2 is abundant, Bax was found to cluster at the membrane surface (Figure 4), and pore formation was inhibited unless the Bcl-2 abundance was low, as seen in Figure 3D. Since the main features for sequestering Bax by Bcl-2 were similar for membrane systems with and without CL, we suggest the main binding point between Bcl-2 and Bax must be located near the bilayer interface.

## Conclusions

The findings presented here provide structural evidence for the cell-protecting mechanism of antiapoptotic members of the Bcl-2 family. Overexpression of Bcl-2 has been found in a number of cancers, where it contributes to disease proliferation by reducing apoptosis. Here, we provide direct molecular-level structural evidence obtained in the membrane environment for the role of Bcl-2/Bax complexation in preventing apoptosis. We found that Bax sequestration may result from not only heterodimeric binding to Bcl-2 but also subsequent binding to itself.

## Materials and Methods

### Materials

#### Lipids

Tail-deuterated 1-palmitoyl-2-oleoyl-d_63_-*sn*-glycero-3-phosphocholine (d-POPC) was synthesized by the Deuteration and Macromolecular Crystallography (DEMAX) platform at the European Spallation Source (ESS), Lund, Sweden, using the published method.^68^ Natural abundance hydrogen 1-palmitoyl-2-oleoyl-*sn*-glycero-3-phosphocholine (h-POPC) and cardiolipin from bovine heart (CL) were purchased from Sigma-Aldrich as solid powders.

##### Expression and Purification of Protonated (h-Bax) and Deuterated (d-Bax) Proteins

Production of the Bax protein for NR, EM, and ATR-FTIR studies was accomplished by following the previously published method.^69^ Deuteration (>90%) of Bax was carried out in a similar fashion, but using a M9 minimal media recipe as 1 L media was prepared by mixing 13 g of KH_2_PO_4_, 10 g of K_2_HPO_4_, 9 g of Na_2_HPO_4_, 2.4 g of K_2_SO_4_, 2 g of NH_4_Cl, 2.5 mL of MgCl_2_ (2.5 M stock), 1 mL of thiamine (30 mg mL^–1^ stock), 2 g of glucose (nondeuterated), and 2 g of NH_4_Cl (nondeuterated), followed by the addition of trace elements (1 mL of 50 mM FeCl_3_, 20 mM CaCl_2_, 10 mM MnCl_2_, 10 mM ZnSO_4_, 2 mM CoCl_2_, 2 mM CuCl_2_, 2 mM NiCl_2_, 2 mM Na_2_MoO_4_, and 2 mM H_3_BO_3_), 100 μg/mL carbenicillin, and 34 μg/mL chloramphenicol. The pH of the medium was adjusted to 6.9 and sterile-filtered before use.

##### Expression and Purification of Protonated (h-)­Bcl-2

The expression and purification of protonated Bcl-2 were carried out as previously reported.^31^ The reconstitution of Bcl-2 into proteoliposomes in POPC (1-palmitoyl-2-oleoyl-*sn*-glycero-3-phosphocholine) and cardiolipin (1,3-bis­(*sn*-3′-phosphatidyl)-*sn*-glycerol) was accomplished by following the method described in literature.^51^ All proteoliposomes were prepared as previously described^32^ to an expected protein:lipid molar ratio of 1:70. The final protein to lipid composition in the formed bilayers is determined from the neutron reflectometry fitting as a volume fraction. The protein concentration of Bax is stated in the Methods section.

Successful incorporation of Bcl-2 protein into the various bilayer systems was verified by SDS-PAGE prior to neutron reflectometry experiments.

### Methods

#### Neutron Reflectometry Measurements

NR measurements were performed on the white beam SURF and OFFSPEC^70^ reflectometers at the ISIS Neutron and Muon Source (Rutherford Appleton Laboratory, Oxfordshire, UK) and on the Figaro reflectometer at the Institut Laue Langevin (ILL, Grenoble, France) using neutron wavelengths from 0.5 to 7, 1 to 14, and 2 to 20 Å. The reflected intensity was measured at glancing angles of 0.35, 0.65, and 1.5° for SURF, 0.7 and 2.0° for OFFSPEC, and 0.7 and 2.3° for Figaro. Reflectivity was measured as a function of the wave vector transfer, *Q*_*z*_ (*Q*_*z*_ = (4π sin θ)/λ, where λ is the wavelength and θ is the incident angle). Data was obtained at a nominal resolution (*dQ*/*Q*) of 3.5% at ISIS and 7.0% at ILL. The total illuminated sample length was ∼60 mm on all instruments. Measurement times for a single reflectometry data set (∼0.01 to 0.3 Å^–1^) were 40 to 180 min at ISIS and 20 to 60 min at ILL. Data collection times for kinetic data sets varied and can be seen as the x-error bar in Figures 3D (inset) and 4D.

Details of the solid–liquid flow cell and liquid-exchange setup used in the experiments described here have been reported by us previously.^71^ Briefly, solid–liquid flow cells containing piranha acid (sulfuric acid, hydrogen peroxide, and water mixture) cleaned 111 silicon substrates (15 × 50 × 80 mm with one 50 × 80 mm surface polished to 3 Å root mean squared roughness) were placed onto the instrument sample position and connected to instrument controlled HPLC pumps (Knauer Smartline 1000), which enabled programmable control of the change of solution isotopic contrast in the flow cell.

##### Vesicle Preparation for NR

POPC vesicles were prepared for deposition by hydrating the lipid in D_2_O to a concentration of 0.2 mg mL^–1^, bath sonicating for 30 min, and tip sonicating on ice for 10 min (1s on 2s off) to produce vesicles of roughly 100 nm diameter. Bcl-2-containing vesicles were prepared by hydrating the pellet in deposition buffer (10 mM sodium citrate pD/H 3.8, 25 mM NaCl, 1.25 mM CaCl_2_), centrifuging back to a pellet while discarding the supernatant, and resuspending the pellet in the deposition buffer to a lipid concentration of 0.2 mg mL^–1^. The Bcl-2-containing vesicles were then tip-sonicated on ice for 10 min (1s on 2s off), to an average diameter of roughly 150–200 nm, ensuring minimal time between sonication and injection into the ATR-FTIR or NR flow cell.

##### Lipid Membrane Deposition

Initially, the clean silicon substrates were characterized by NR in D_2_O and H_2_O buffer solutions. Then, freshly sonicated lipid vesicle solutions (0.2 mg mL^–1^) were introduced into the cells in the experiment deposition buffer solution of 10 mM sodium citrate pD/H 3.8 25 mM NaCl 1.25 mM CaCl_2_ or 20 mM HEPES pD 7.2 150 mM NaCl 2 mM CaCl_2_. The samples were incubated at 30 ± 1 °C for ∼1 h before the nonsurface-bound vesicles were removed by flushing the cells with 15 mL (∼5 cell volumes) of the same buffer solution, before a solution of pure D_2_O was flushed into the cell. This led to the formation of high-quality (i.e., high-coverage) supported lipid bilayers at the solid/liquid interface. The resulting bilayers were characterized by NR in the experiment buffer (20 mM sodium phosphate, pH 7.4, 50 mM NaCl, 1 mM EDTA) under four solution isotopic contrast conditions of D_2_O, 80% D_2_O: 20% H_2_O, silicon-matched water (Si-MW, 38% D_2_O), and H_2_O buffer solutions. The remainder of the beamtime was conducted in this buffer.

##### Bax Interaction

Once the characterization of the SLB was complete, the sample’s surface was placed in the correct experiment buffer isotopic contrast (D_2_O for h-proteins and H_2_O for d-proteins) and ∼6 mL of a 0.1 mg mL^–1^ Bax solution was injected into the flow cell (the cell volume is 3 mL) either by hand (SURF) or using a syringe pump (OFFSPEC and INTER, AL1000–220, World Precision Instruments; Figaro, The Harvard Apparatus Pump 33 DDS). In most cases, the interaction of the protein with the SLB was monitored by NR, with data sets collected continuously until an equilibrium interaction between the protein and the SLB was verified by no further changes in the data being observed against time. At this point, a final equilibrium data set was collected; then, the excess protein was flushed from the cell, and the structure of the surface protein–lipid complex was examined by NR under three solution isotopic contrast conditions (D_2_O, Si-MW, and H_2_O). It should be noted that no difference was found in any sample between the equilibrium Bax bound data before and after flushing of the excess protein, suggesting that the protein–lipid complexes formed at the sample surface were stable.

##### NR Data Analysis

NR data was analyzed using the RasCal software (A. Hughes, ISIS Spallation Neutron Source, Rutherford Appleton Laboratory), which employs optical matrix formalism^72^ to fit layered models of the structure across bulk interfaces and allows for the simultaneous analysis of multiple NR data sets collected under different sample and isotopic contrast conditions and permits them to be fully or partially constrained to the same surface structure in terms of thickness profile but vary in terms of neutron scattering length density. For additional details, see Supporting Information Section 1.

## Supplementary Material



## Data Availability

NR data and custom model scripts used in NR data fitting, as well as ATR-FTIR data, are available via 10.5281/zenodo.14698607. Information on the characterization and purification of tail-deuterated POPC can be found at 10.5281/zenodo.4160419 and 10.5281/zenodo.14002732.

## Notes

Section 1 provides a detailed description of the experimental methods used in this study. Section 2 provides a description of the interaction of Bax with a POPC-only SLB by NR used as a control measurement for comparison with the interaction of Bax with POPC::Bcl-2 SLBs (Figure S1). In addition, this section also contains detailed figures of the NR data and model-data fits used to resolve the component volume fraction vs distance profiles shown in Figure 3 (Supporting Information Figures S2–S6 and Tables S1–S4). The structural parameters derived from the model-data fitting are given in Supporting Information Table S5. ATR-FTIR data from the interaction of Bax with a POPC SLB with and without Bcl-2 are shown in Supporting Information Figures S7 and S8, respectively. Selected cropped EM images of d-POPC: h-Bcl-2 vesicle before and after the interaction of Bax are shown in Supporting Information Figure S10, with the EM images from which these were selected being shown in full in Supporting Information Figures S11 and S12. Section 3 describes comparative modeling of a series of Bax monomers and multimers and the radius and diameters of gyration obtained from these, which were compared with the oligomeric distributions found by NR described here (PDF)

## ○

Institut Laue Langevin, Grenoble 38042, France
