# Supplementary material for: Avoiding Mitochondrial Apoptosis by the Bcl-2-Driven Bax Oligomerization on Membrane Surfaces
Source: ACS Chem Biol. 2026 Feb 18;21(3):565–76. doi: 10.1021/acschembio.5c00913 (PMC13010249; doi:10.1021/acschembio.5c00913)
Supplement: Supplementary file 1 [file cb5c00913_si_001.pdf]

Supporting Information

Avoiding Mitochondrial Apoptosis by Bcl-2 driven Bax Oligomerization on Membrane Surfaces

Sophie E. Ayscough[1,2,3,+] Luke A. Clifton[1, *], Jörgen Ådén[4], Sebastian Köhler [5], Nicolò Paracini[2,6], James Doutch[1], Éilís C Bragginton [7], Anna. E. Leung[2], Oliver Bogojevic[2], Jia-Fei Poon[2], Tamás Milán Nagy[4], Hanna P. Wacklin-Knecht[2,3], Gerhard Gröbner[4, *]

[1] ISIS Pulsed Neutron and Muon Source, Science and Technology Facilities Council, Rutherford Appleton Laboratory, Harwell Science & Innovation Campus, Didcot, Oxfordshire, OX11 OQX, UK.

[2] European Spallation Source ERIC, ESS, P.O. Box 176, SE-22100 Lund, Sweden.

[3] Department of Chemistry, Division of Physical Chemistry, Lund University, P.O. Box 124, SE-22100 Lund, Sweden

[4] Department of Chemistry, University of Umeå, SE -901 87, Umeå, Sweden

[5] Lund Institute for Neutron and X-ray Scattering, Department of Chemistry, Lund University, P.O. Box 124, SE-22100 Lund, Sweden

[6] Institut Laue Langevin, 38042, Grenoble, France

[7] Electron Bio-Imaging Centre (eBIC), Diamond Light Source Ltd, Diamond House, Harwell Science and Innovation Campus, OX11 0DE, UK.

* Luke A. Clifton: luke.clifton@stfc.ac.uk *Gerhard Gröbner: gerhard.grobner@chem.umu.se

[+]Current affiliation: Institut Laue Langevin, 38042, Grenoble, France

Section 1: Additional Methods

Neutron Reflectometry Data Analysis:

Neutron Reflectivity data was fitted with the Rascal 2.0 package [1], using the custom model option whereby the fitted parameters defining the interfacial layers are given definitions and constraints. The structure of the substrates, the bilayers and the bilayer-protein interactions were determined by finding the parameters that minimized the chi^2 value between the reflectivity data and the fitting model. The bilayers were fitted as 5-layer model, from the substrate to the subphase; a thin SiO_2 layer, an inner lipid head group layer, an inner tail group layer, an outer tail layer and an outer head group layer. The molar ratio of head to tail groups of the lipids were maintained by fitting a lipid area per molecule parameter, thickness of the bilayer head t_{hg} and tail regions t_t are given by:

$$t_{hg} = \frac{V_{hg} + n_w V_w}{APM}$$

and

$$t_t = \frac{V_t}{APM} ,$$

Where V_{hg} , V_t and V_w are the average volumes of the head and tails groups and that of a water molecule respectively, n_w is the number of water molecules per lipid head and APM is the lipid area per molecule. In all the fits of this publication the APM and n_w are constrained to be the same for both bilayer leaflets. The scattering length density of a component is defined by:

$$SLD = \frac{\sum b}{V},$$

Where $\sum b$ is the sum of the component scattering lengths and V is the molecular volume of the component. The molecular volumes and scattering length densities of the lipid heads and tails were calculated from literature values[2], addition of their sub-component volumes and addition of their -component scattering lengths, full detail in the NR scripts included in our electronic supplementary information. The SLD of each layer is calculated by fitting the volume fraction of each component in that layer. In addition to the water per lipid heads parameter allowing for a volume fraction of water in the more hydrophilic head layer, we have a hydration parameter across the entire bilayer, both head and tail layers, to allow for any patchiness of the bilayers. For the bilayers containing the integral membrane protein Bcl-2, a single volume fraction (VF) of Bcl-2 is fitted across the bilayer layers. Each of the interfaces between layers in the model have an associated roughness, we allow a separate roughness to be fitted for the Si-SiO_2 interface SiO_2-bilayer interface, otherwise the bilayer interface roughness's are constrained to be the same fitted value.-bilayer interface, otherwise the bilayer interface roughness's are constrained to be the same fitted value.

For Bcl-2 containing bilayers on equilibrium binding of Bax, with exception of the POPC:CL bilayer with low Bcl-2 VF of about 5%, a seven-layer model was found to be the most reasonable model that satisfactorily resolved the features in the data. The first 5 layers

were the thin SiO_2 layer and bilayer, the APM, for which the hydration and water per lipid head parameters were re-fitted after the Bax interaction but the Bcl-2 volume fraction was assumed to not change and was constrained to be the same as before Bax interaction using:

$$VF_{Bcl-2_{ab}} = VF_{Bcl-2_{bb}} \times \left(\frac{t_{b_{bb}}}{t_{b_{ab}}}\right),$$

Where $VF_{Bcl-2_{bb}}$ is the volume fraction of Bcl-2 in the bilayer prior to Bax interaction, t_bi. s the bilayer thickness before (bb) and after (ab) Bax interaction. This is done as we assume the amount of Bcl-2 at the interface will not change. We also allow for a volume fraction of Bax across the bilayer, although there was significantly less Bax insertion than in the lipid only data sets. The two added layers are protein layers, where the volume fraction of Bax and the thickness of the layers are fitted.

For the equilibrium binding of Bax to the POPC bilayers without Bcl-2, a similar model was used as previously for POPC:CL bilayers (*2*). This model is an eight-layer model of a disrupted bilayer and a lipid protein complex. The first five layers (SiO_2 + lipid bilayer) of the structure were the same as prior to the Bax interaction, with an increase in protein and water content and a decrease in the lipid content. The three added layers are a Bax-protein only layer next to the bilayer, a mixed Bax-protein/lipid layer and an additional Bax-protein layer adjacent to the bulk solution. A similar model was used to fit the POPC:CL bilayer with a low volume fraction of Bcl-2 (5%), in which lipid removal was also observed. The Bcl-2 was constrained, but the bilayer disruption was modeled as a decrease in lipid and increase in water content and 3 additional layers on top of the bilayer consisting of a Bax-protein only layer next to the bilayer, a mixed Bax-protein/lipid layer and an additional Bax-protein layer adjacent to the bulk solution.

Analysis of time-resolved (TR)-NR data

The TR-NR data describing the Bax interaction with a d-POPC: Bcl-2 bilayer, was measured in a single subphase contrast (D_2O) after the injection of Bax into the NR flow cell. This data was batch-fitted. The parameters of the final equilibrium Bax-bilayer fit were used as a fixed constraint, allowing for only two parameters of the Bax membrane surface component to vary. The best-fit of the TR-NR datasets was found allowing for the value of

the volume fraction of Bax and surface layer thickness to vary, the maximum volume fraction of Bax being that found in the equilibrium fit. The calculated volume fraction value was applied at the same value to the volume fraction of Bax inside the bilayer, in the first membrane associated Bax layer and then the second Bax layer. Similarly, the thickness value was applied to the first and second Bax layers. Table S.4 shows the fit values and how this relates to the thickness and volume fraction parameters in the model.

NR Error Estimation and plotting

Bayesian analysis was used to calculate the confidence intervals of the neutron reflectivity model to data fitting parameters and therefore provide the error estimation for our calculated structures. Bayesian analysis was done in Rascal using Monte-Carlo-Markov Chain (MCMC) and Delayed-Rejection Adaptive Metropolis (DRAM) algorithms routines to refit the data from the already chi2 minimized fit (3). The parameter uncertainties are determined from the posterior distributions as the 65% confidence interval and the uncertainties on the scattering length density and reflectivity plots were generated from 1000 random samples from the Markov chains. These chain samples are used to generate the line shading in the reflectivity, SLD and volume fraction plots whilst the darker lines represent the mean fit lines.

Component Volume Fraction Profiles

Volume fraction profiles detailing the distribution of components across the solid/liquid interface before and after the equilibrium interaction of Bax were produced using a bespoke script. MCMC Bayesian error estimation results and the relationship between the fitting parameters and interfacial structure in the RasCal custom model were used to determine the distribution of each structural component in the volume fraction vs. distance profile. The volume fraction of an individual component was calculated in 1 Å increments across the solid/liquid interface (the silicon/silicon dioxide interface set as zero). The mean, lower, and upper 65% confidence interval bounds of each component distribution were determined for every 1 Å segment using the MCMC error estimation results or derived parameters; these confidence intervals were then used to produce a line width error region above and below the mean values. The water distribution was calculated as the remaining unoccupied volume for each 1 Å slice and summed across the interface with the appropriate error propagation.

CryoEM sample preparation and data collection

Two 100 µL aliquots of Bcl-2 containing POPC vesicles were prepared at a protein concentration of 0.5 mg ml^{-1} by resuspending pellets in buffer (20 mM Sodium Phosphate, pH 7.4, 50 mM NaCl, 1mM EDTA) and tip sonicating to an average diameter of about 200 nm. To one aliquot, 100 µl of 0.1 mg ml^{-1} Bax was added and the samples incubated at 37° C for 1 hour. They were then frozen within 30 minutes.

Four microliters of freshly prepared proteoliposomes at a Bcl-2 protein concentration of 0.5 mg/ml were deposited onto glow discharged R1.2/1.3 Cu 300 mesh holy carbon grids (Quantifoil) prior to vitrification. Grids were plunge frozen using a Virtobot Mark IV (Thermo Fisher Scientific) (Blot force 2, Blot time 3 sec, temperature 22 degrees) and stored in liquid nitrogen prior to imaging. Data was collected at the electron Bio-Imaging Centre (eBIC) using a Titan Krios microscope (Thermo Fisher Scientific) equipped with a field emission gun operating at 300 keV, a Falcon 4i direct electron detector with a Selectris X imaging filter (Thermo Fisher Scientific). Data was collected with EPU software (Thermo Fisher Scientific) at a magnification of x130 000 with a corresponding pixel size of 0.921 Å/pixel. The total dose applied to the sample was 40 e-/Å^2.

Attenuated Total Reflection Fourier Transform Infra-Red Spectroscopy (ATR-FTIR)

Trapezoidal silicon substrates for attenuated total reflection infrared spectroscopy were obtained from Crystran (Poole, UK). These substrates were made to fit into a Specac (Orpington, UK) liquids ATR accessory which was, itself, fitted into the sample cavity Thermo-Fisher iS50 Infra-red spectrometer (Waltham, MA, USA). The substrates have four polished faces (to ~6 Å root mean squared roughness) the largest being a 72 mm × 10 mm face which was used as the sample surface. The IR beam enters the substrate through a polished face at 45° relative to the sample surface, total internal reflectance of the IR beam inside the substrate gives rise to six evanescent waves on the sample surface. IR spectra were collected at a resolution of 4 cm^{-1}.

To reduce the influence of water vapour on the IR spectra the iS50 instrument and ATR mirror assembly accessory was continuously purged with dry air from a Peak scientific (Glasgow, UK) CO_2 and water removing air purge. The Specac liquid ATR cell was

modified to fit Omni-fit tubing which was connected to a syringe pump (AL1000-220, World Precision Instruments, Hitchin, UK). The experimental D_2O buffer solution (20 mM Sodium Phosphate, pD 7.4, 50 mM NaCl, 1mM EDTA) was injected into the ATR flow cell which was then heated to 30±1°C using a water bath (Julabo, Seelbach Germany). D_2O solutions were used for all ATR-FTIR measurements as the D_2O bending mode is lower (1215 cm^{-1}) compared to H_2O (1645 cm^{-1}) meaning limited contamination of protein amide I region by spectral bands from water (4). After buffer flushing a background spectrum was collected. Spectra were then collected monitoring the removal of water vapor from the spectrometer and mirror assembly until a steady state was reached. A 2nd background measurement was then collected before deposition of the SLB.

SLB deposition was conducted using the same methodology as described for NR measurements and monitored through the appearance of CH_3 / CD_3 asymmetric, CH_2 / CD_2 asymmetric and CH_2 / CD_2 symmetric stretches from the lipid tails at ~2950 cm^{-1}, 2920 cm^{-1} and 2850 cm^{-1} respectively and the appearance of a lipid carbonyl stretch at 1730 cm^{-1}. In the case of the d-POPC: h-Bcl2 sample an amide I peak from the Bcl-2 carbonyls (predominantly) was also observed during SLB fabrication. Once the SLB was formed Bax was injected into the solid/liquid flow cell and its accumulation at the near surface region was monitored through the appearance of a protein Amide I peak.

Section 2: Additional Information

POPC Only control measurement

Bax forms pores in POPC only membranes: Control NR measurements on basic POPC bilayers revealed that Bax induced pore formation into the SLB through lipid removal and redistribution of this into protein-lipid clusters on the membrane surface (Fig S1). The final POPC-Bax dataset was obtained at 30ºC upon equilibrium Bax interaction (i.e. when no further time dependent changes were observed in the NR data). This mechanism was similar, but significantly slower (see Fig S9), than that previously observed by us in the presence of CL containing target membranes[5]. However, a higher volume fraction of Bax was found to embed within the hydrophobic core of the POPC lipid bilayer compared to its interaction with CL containing membranes. Additionally, our previous results showed that lipid removed from the CL containing MOM-models was completely redistributed to the membrane surface in Bax-lipid clusters. For the Bax interaction with the POPC only membrane this was only partially found with a ~20% loss of lipids (see Table 1) from the bilayer during Bax induced pore formation but only around 25% of that removed lipid appeared in Bax lipid complexes found on the membrane surface, suggesting the other 75% of the removed lipids moved into the bulk solution possibly as solution stable Bax-POPC clusters.

As summarized in Table 1, the POPC only bilayers displayed a surface coverage of nearly 100 ±1 % lipid, a value reduced to 82 ± 2 % upon addition of h-Bax. The water content in the membrane hydrophobic core increased from 0 to 2.9 ± 1.4 volume %, reflecting the Bax induced membrane perforation. Even the bilayer thickness decreased by ca. 2 Å, a similar change as observed for CL containing bilayers upon Bax induced pore formation (*14*). The obtained volume fraction values for Bax protein and water in the POPC bilayers support significant membrane perforation by Bax similar as observed with CL containing bilayers which are closer to MOM-like systems[5,6,7]. While in those membranes CL drives activation of Bax into pore forming oligomers in a faster CL dependent way, here in POPC this activation occurs on a slower hourly time scale, with a time constant of Bax-binding of 739 +/- 22 min, see ATR-FTIR analysis in Fig S7 and Fig S9, vs 175 +/- 15 min for 10% CL bilayers[5]. However, the Bax mediated pore formation mechanism appears consistent This clearly shows that direct activation of Bax by lipids alone does not rely on the presence of anionic lipids[8,9,10] but can take place on neutral bilayers containing zwitterionic

lipids, albeit on a slower time scale of several hours. This activation must include the conversion of inactive soluble Bax monomers to membrane-associated active Bax monomers[9,11,12,13]. The interaction with the lipids then drives the conversion of the Bax via its α2-α5 core domain into active dimers causing further assembly into 6-10 monomer large subunits and into pores[7,14,15], and finally releasing apoptotic factors, causing irreversible cell death. Assembly of additional Bax into multimeric states can further increase pore size by a kind of organized clustering near membrane-rupturing pores[5,16,17].

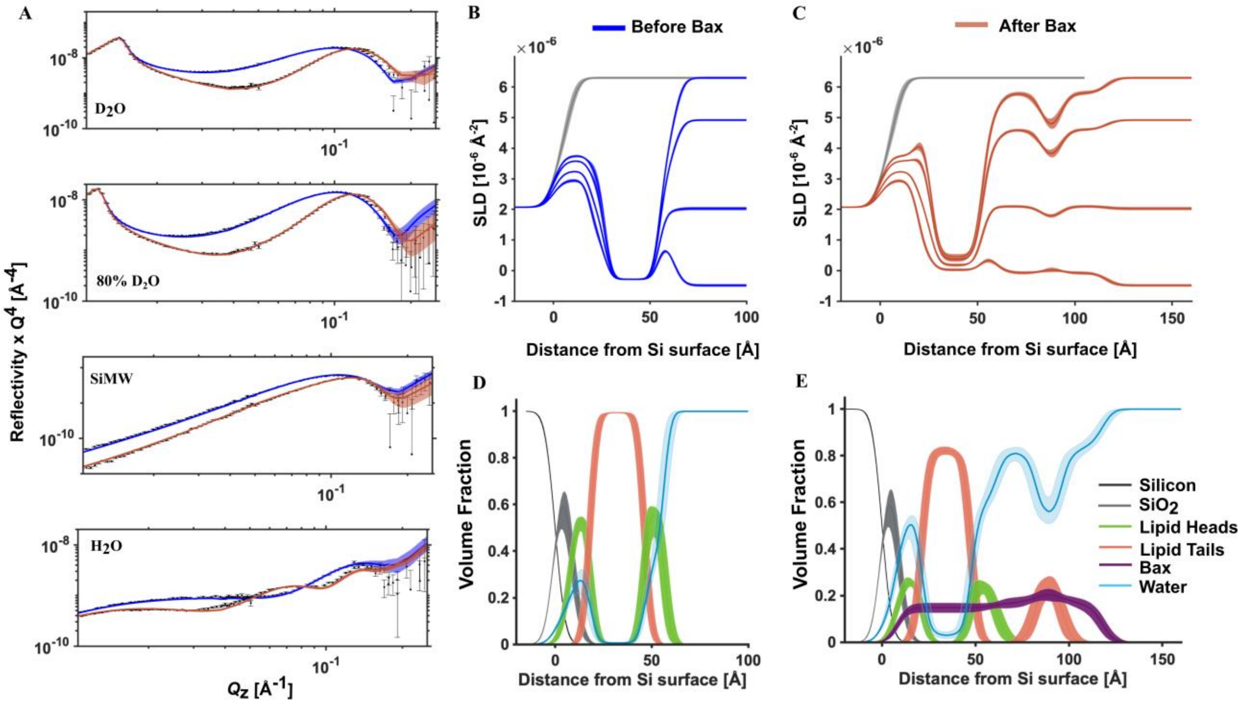

Figure S1. NR data showing Bax-induced pore formation in a POPC only bilayer. NR data (error bars) and model data fits (lines, χ^2 = 101.0; see also supplement Table S1) from a natural-abundance hydrogen POPC (h-POPC) SLB before (blue) and after (red) the interaction of (h-)Bax are shown in four differing solution isotopic contrast conditions being D_2O, 80% D_2O, silicon-matched water (Si-MW) and H_2O buffer solutions (A). The scattering length density (SLD) profiles are shown for the surface structure before (blue, B) and after (red, C) the h-Bax interaction, the bare substrate SLD profile is also shown (grey, B and C). The corresponding component volume fraction profiles are shown before (D) and after (E) the h-Bax interaction as determined from the NR fits. Individual components are color-coded as indicated, with the Bax protein distribution in purple. Note that after the interaction of the protein there is a lower lipid content in the SLB and a new distribution of lipid on the membrane surface. Line widths in the NR data fits represent the 65% confidence interval of the range of acceptable fits determined from Monte-Carlo-Markov Chain (MCMC) error analysis and the line widths in the SLD and volume fraction profiles represent the ambiguity in the resolved interfacial structure determined from these.

**Table S1: The resolved structural components before and after the interaction of Bax with SLB composed of POPC and POPC containing Bcl-2 protein. Bax Surface layer composition described as layers numbered outwards from the membrane, where 1 is membrane adjacent. ***Values in parentheses represent the 65% confidence intervals determined from MCMC resampling of the experimental data fits.

	Average Area per Lipid Molecule / Å^2	Tails Thickness / Å	Tails Composition	Head Group Thickness/ Å	Head-group Composition	Membrane Surface BAX/Lipid Complex Thickness / Å	BAX Surface Layer Composition
**POPC**	66.0 Å^2 (65.7 Å^2, 66.4 Å^2)	28.2 Å (28.1 Å, 28.3 Å)	Lipid 100% (99.5%, 100%) Solution 0% (0%, 0.5%)	7.4 Å (6.9 Å ,8.0 Å)	Lipid 67.5% (63.1%, 72.8%) Water 32.5% (27.2%, 36.9%)	-	-
**POPC + h-BAX**	70.9 Å^2 (69.4 Å^2, 72.6 Å^2)	26.3 Å (25.7 Å, 26.9 Å)	Lipid 82.2% (80.6%, 84.1%) Bax Protein 14.9% (12.6%, 16.8%) Solution 2.9% (1.5%, 4.3%)	13.3 Å (11.5 Å, 14.5 Å)	Lipid 28.9% (26.3%, 34.4%) Bax Protein 14.9% (12.6%, 16.8%) Solution 55.7% (52.0%, 58.5%)	**1**, 22.5 Å (20.8 Å, 24.0 Å) **2**, 11.6 Å (9.0 Å, 15.1 Å) **3**, 22.5 Å (20.8 Å, 24.0 Å) **Total:** 56.6 Å (54.8 Å, 58.8 Å)	1, Protein 16.7% (14.0%, 19.3%) Solution 83.3 % (80.7%, 86.0%) 2, Protein 23.0 % (17.4%, 27.0%) Lipid 10.3% (5.7%, 14.6%) Solution 67.5% (60.9%, 74.1%) 3, Protein 16.7% (14.0%, 19.3%) Solution 83.3 % (80.7%, 86.0%)
**d-POPC: Bcl-2**	76.5 Å^2 (69.1 Å^2 84.2 Å^2)	24.4 Å (22.0 Å 27.0 Å)	Lipid 48.6% (41.6% 55.7%) Bcl-2 protein 39.9% (36.6% 43.3%) Solution 11.5% (9.4% 13.6%)	9.3 Å (7.6 Å 10.9 Å)	Lipid 22.5% (21.2% 24.0%) Bcl-2 protein 39.9% (36.6% 43.3%) Solution 37.6% (31.3% 42.4%)	-	-
**d-POPC: Bcl-2 + h-BAX**	81.3 Å^2 (73.0 Å^2 89.6 Å^2)	22.9 Å (20.8 Å 25.6 Å)	Lipid 46.0% (38.0% 52.8%) Bcl-2 protein 37.3% (33.2% 42.1%) Bax protein 9.4% (4.0% 14.7%) Solution 4.5% (1.4% 8.3%)	8.8 Å (7.0 Å 10.7 Å)	Lipid 22.0% (18.1% 27.7%) Bcl-2 protein 37.3% (33.2% 42.1%) Bax protein 9.4% (4.0% 14.7%) Solution 30.3% (25.8% 33.4%)	**1,** 55 Å (50.6 Å 58.8 Å) **2,** 52.8 Å (24.5 Å 76.9 Å) **Total:** 108 Å (74 Å 134 Å)	**1,** Protein 26.2% (23.2% 29.1%) Solution 73.8% (70.9% 76.8%) **2,** Protein 4.5% (2.4% 9.2%) Solution 95.5% (90.8% 97.6%)

Table S2. Scattering length densities of system components used in the models for fitting and understanding of the neutron reflectivity data. Given cardiolipins low volume fraction in the membrane and the mix of tails in the heart CL used, we used the SLD of h-POPC tails only in our analysis. *Value for 100% deuterated tails given, in our models the actual SLD assumes 95% deuteration. Bax and Bcl-2 values were obtained from the Biomolecular Scattering Length Density Calculator(5).

Component	Scattering Length Density (in H_2O) $\times 10^{-6}$ Å^{-2}	Scattering Length Density (in D_2O) $\times 10^{-6}$ Å^{-2}
POPC lipid head group	1.81	1.81
h-POPC lipid tails	-0.29	-0.29
d-POPC lipid tails	6.85	6.85
Cardiolipin lipid head	4.21	4.52
Bcl-2	1.90	3.20
Bax	1.85	3.05
d-Bax	5.87	7.60

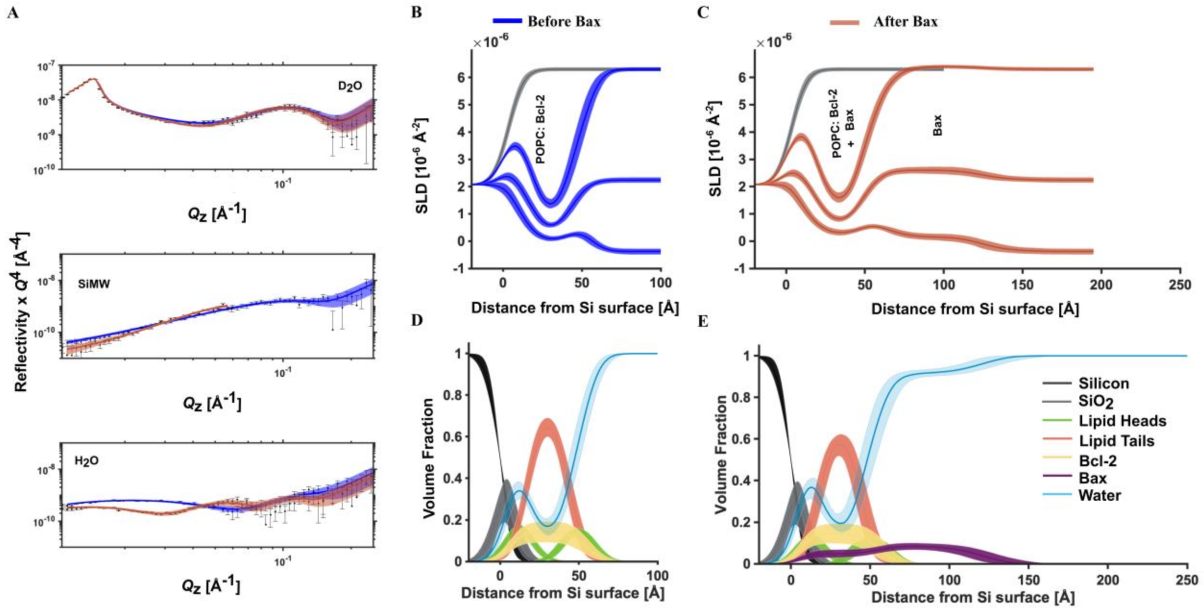

Figure S2. NR data showing d-Bax binding to a h-POPC Bcl-2 lipid bilayer. NR data (error bars) and model data fits from a h-POPC (lines, χ^2 = 19.7): h-Bcl-2 SLB before (blue) and after (red) the interaction of deuterated (d-)Bax are shown in three differing solution isotopic contrast conditions being D_2O, Si-MW and H_2O (A) buffer solutions. The scattering length density (SLD) profiles are shown for the surface structure before (B) and after the h-Bax interaction (C). The corresponding component volume fraction profiles are shown before (D) and after (E) the h-Bax interaction as determined from the NR fits. Individual components are color-coded as indicated, with the Bcl-2 distribution in orange and the Bax protein distribution in purple. Line widths in the NR data fits represent the 65% confidence interval of the range of acceptable fits determined from Monte-Carlo-Markov Chain (MCMC) error analysis and the line widths in the SLD and volume fraction profiles represent the ambiguity in the resolved interfacial structure determined from these.

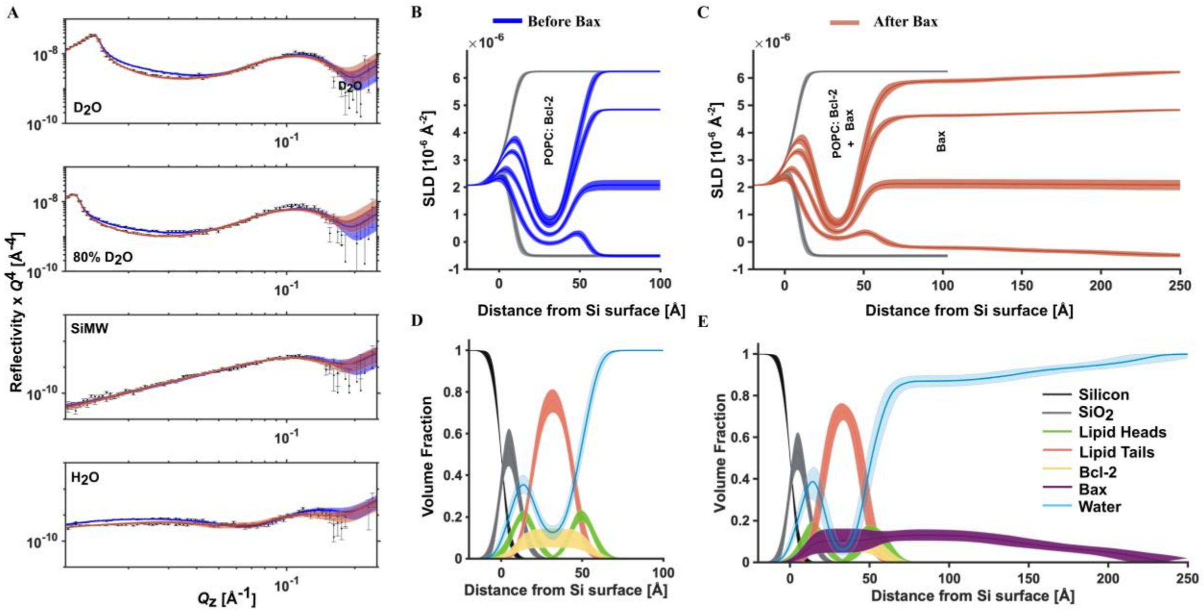

Figure S3. NR data showing h-Bax binding to a h-POPC Bcl-2 lipid bilayer. NR data (error bars) and model data fits (lines, X^2 = 46.2; see also supplement Table 1) from a h-POPC/Bcl-2 SLB before (blue) and after (red) the interaction of natural abundance hydrogen (h-)Bax are shown in four different solution isotopic contrast conditions being D_2O, 80% D_2O, Si-MW and H_2O (A) buffer solutions. The scattering length density (SLD) profiles are shown for the surface structure before (B) and after the h-Bax interaction (C). The corresponding component volume fraction profiles are shown before (D) and after (E) the h-Bax interaction as determined from the NR fits. Individual components are color-coded as indicated, with the Bcl-2 distribution in orange and the Bax protein distribution in purple. Line widths in the NR data fits represent the 65% confidence interval of the range of acceptable fits determined from Monte-Carlo-Markov Chain (MCMC) error analysis and the line widths in the SLD and volume fraction profiles represent the ambiguity in the resolved interfacial structure determined from these.

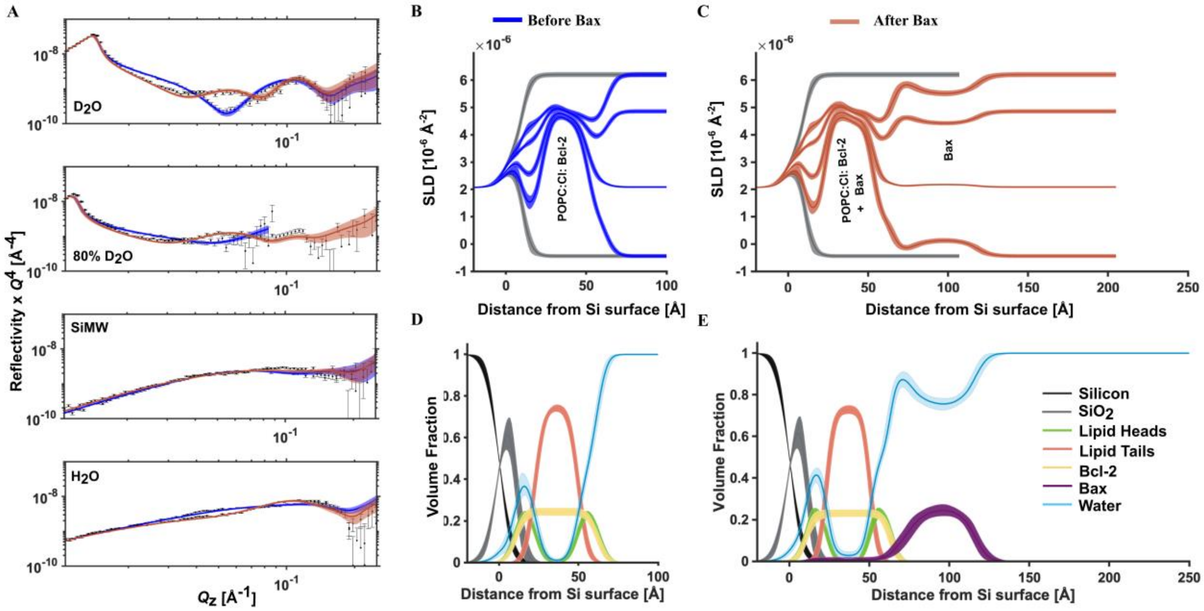

Figure S4. NR data showing h-Bax binding to a d-POPC cardiolipin (9:1) Bcl2 lipid bilayer. NR data (error bars) and model data fits (lines, χ^2 = 46.2) from a d-POPC/Bcl-2 SLB before (blue) and after (red) the interaction of natural abundance of hydrogen (h-)Bax are shown in four differing solution isotopic contrast conditions being D_2O, Au-MW, Si-MW and H_2O (A) buffer solutions. The scattering length density (SLD) profiles are shown for the surface structure before (B) and after the h-Bax interaction (C). The corresponding component volume fraction profiles are shown before (D) and after (E) the h-Bax interaction as determined from the NR fits. Individual components are color-coded as indicated, with the Bcl-2 distribution in orange and the Bax protein distribution in purple. Line widths in the NR data fits represent the 65% confidence interval of the range of acceptable fits determined from Monte-Carlo-Markov Chain (MCMC) error analysis and the line widths in the SLD and volume fraction profiles represent the ambiguity in the resolved interfacial structure determined from these.

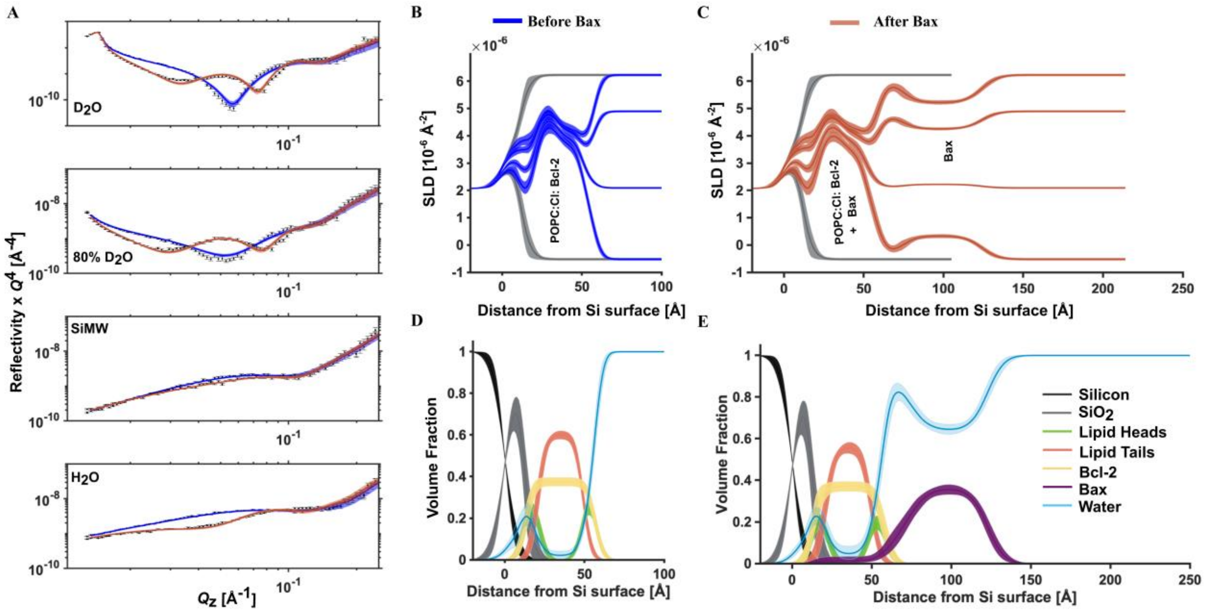

Figure S5. NR data showing h-Bax binding to a d-POPC h-cardiolipin (9:1) Bcl-2 lipid bilayer. NR data (error bars) and model data fits (lines, X^2 = 34.5) from a d-POPC:CL/Bcl-2 SLB before (blue) and after (red) the interaction of natural abundance of hydrogen (h-)Bax are shown in four different solution isotopic contrast conditions being D_2O, Au-MW, Si-MW and H_2O (A) buffer solutions. The scattering length density (SLD) profiles are shown for the surface structure before (B) and after the h-Bax interaction (C). The corresponding component volume fraction profiles are shown before (D) and after (E) the h-Bax interaction as determined from the NR fits. Individual components are color-coded as indicated, with the Bcl-2 distribution in orange and the Bax protein distribution in purple. Line widths in the NR data fits represent the 65% confidence interval of the range of acceptable fits determined from Monte-Carlo-Markov Chain (MCMC) error analysis and the line widths in the SLD and volume fraction profiles represent the ambiguity in the resolved interfacial structure determined from these.

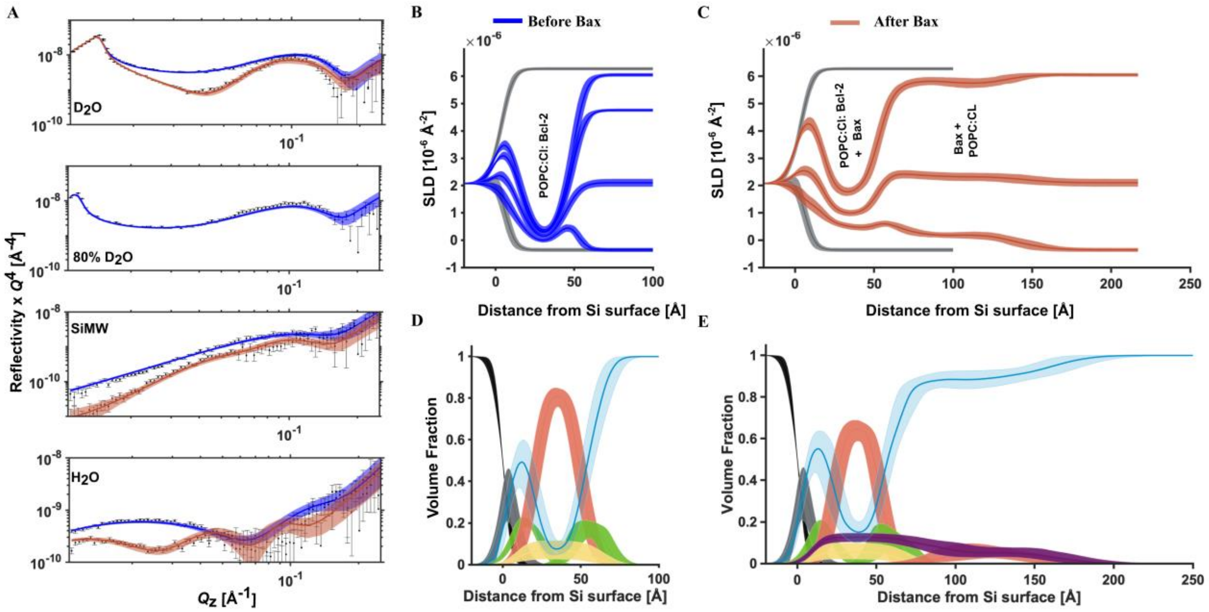

Figure S6. h-POPC:CL (9:1) Bcl-2 bilayer interaction with h-Bax, at low VF of Bcl-2-lipid removal and poration of the membrane still occurs. NR data (error bars) and model data fits (lines, χ^2 = 36.7) from a h-POPC:Cl/Bcl-2 SLB before (blue) and after (red) the interaction of natural abundance of hydrogen (h-)Bax are shown in different solution isotopic contrast conditions being D_2O, Au-MW, Si-MW and H_2O (A) buffer solutions. The scattering length density (SLD) profiles are shown for the surface structure before (B) and after the h-Bax interaction (C). The corresponding component volume fraction profiles are shown before (D) and after (E) the h-Bax interaction as determined from the NR fits. Individual components are color-coded as indicated, with the Bcl-2 distribution in orange and the Bax protein distribution in purple. Line widths in the NR data fits represent the 65% confidence interval of the range of acceptable fits determined from Monte-Carlo-Markov Chain (MCMC) error analysis and the line widths in the SLD and volume fraction profiles represent the ambiguity in the resolved interfacial structure determined from these.

Table S3: The resolved structural components before and after the interaction of Bax with SLBs composed of POPC-cardiolipin containing Bcl-2 protein.

*Values in parentheses represent the 65% confidence intervals determined from MCMC resampling of the experimental data fits.

		Lipid Area per Molecule / Å^2	Tails Thickness / Å	Tails Composition	Head Group Thickness / Å	Outer Head-group Composition	Membrane Surface Bax/Lipid Complex Thickness / Å	Bax Surface Layer Composition
**i) h-POPC: CL: Bcl-2**		65.7 (63.1 68.4)	28.3 (27.3 29.6)	Lipid 93.0 (88.1 97.2)<br>Bcl-2 protein 6.9 (2.7 11.8)<br>Solution 0.1 (0.0 0.1)	6.8 (5.6 8.4)	Lipid 68.5 (55.7 82.7)<br>Bcl-2 protein 6.9 (2.7 11.8)<br>Solution 23.6 (9.8 37.0)		
	**+ d-Bax**	53.3 (51.0 57.6)	35.0 (32.4 36.6)	Lipid 68.6 (64.6 73.0)<br>Bcl-2 protein 5.7 (2.2 10.0)<br>Bax protein 12.6 (10.3 14.6)<br>Solution 11.5 (6.8 15.7)	14.4 (9.6 13.3)	Lipid 29.9 (22.3 44.2)<br>Bcl-2 protein 5.7 (2.2 10.0)<br>Bax protein 12.6 (10.3 14.6)<br>Solution 50.7 (38.1 58.8)	**1,** 34.2 (20.5 50.2)<br>**2,** 42.1 (28.9 52.6)<br>**3,** 34.2 (20.5 50.2)<br>**Total:** 109 (87 136)	**1,** Protein 4.6 (1.8 7.8)<br>Solution 95.4 (92.2 98.2)<br>**2,** Protein 8.9 (6.1 11.6)<br>Lipid 24.3 (20.1 27.6)<br>Solution 67.2 (62.4 72.4)<br>3, Protein 4.6 (1.8 7.8)<br>Solution 95.8 (92.2 98.2)
**ii) d-POPC: Cl: Bcl-2**		67.8 (65.6 69.9)	27.5 (26.7 28.4)	Lipid 73.3 (71.2 75.3)<br>Bcl-2 protein 24.7 (23.0 26.4)<br>Solution 1.7 (0.5 3.6)	10.7 (9.1 11.8)	Lipid 33.5 (30.3 39.4)<br>Bcl-2 protein 24.7 (23.0 26.4)<br>Solution 41.7 (35.3 45.6)		
	**+ h-Bax**	65.2 (63.4 67.4)	28.6 (27.7 29.4)	Lipid 72.5 (69.9 75.0)<br>Bcl2 protein 23.4 (23.0 26.4)<br>Bax protein 0.8 (0.2 1.9)<br>Solution 1.3 (0.2 3.2)	12.7 (12.3 13.0)	Lipid 32.6 (29.5 38.4)<br>Bcl-2 protein 23.4 (23.0 26.4)<br>Bax protein 0.8 (0.2 1.9)<br>Solution 43.1 (36.8 46.5)	**1,** 15.3 (9.2 23.5)<br>**2,** 38.5 (29.2 46.8)<br>**Total:** 55 (50 59)	**1,** Protein 7.8 (2.3 14.4)<br>Solution 92.3 (85.6 97.7)<br>**2,** Protein 24.6 (21.3 28.0)<br>Solution 75.4 (72.0 78.7)
**iii) d-POPC: Cl: Bcl-2**		65.0 (62.1 68.1)	28.7 (27.3 30.1)	Lipid 59.0 (56.9 61.2)<br>Bcl-2 protein 38.7 (36.8 40.5)<br>Solution 2.0 (0.58 4.14)	7.1 (6.5 7.9)	Lipid 42.1 (37.8 46.5)<br>Bcl-2 protein 38.7 (36.8 40.5)<br>Solution 19.2 (14.4 24.0)	-	-
	**+ h-Bax**	67.2 (62.6 68.1)	28.6 (25.8 29.8)	Lipid 56.0 (52.0 59.8)<br>Bcl-2 protein 39.0 (36.2 41.6)<br>Bax protein 1.2 (0.3 3.2)<br>Solution 4.4 (3.1 5.9)	7.3 (6.2 8.5)	Lipid 37.4 (31.9 44.3)<br>Bcl-2 protein 39.0 (36.2 41.6)<br>Bax protein 1.2 (0.3 3.2)<br>Solution 21.6 (15.0 27.3)	**1,** 17.6 (13.8 22.2)<br>**2,** 46.8 (40.4 53.1)<br>**Total:** 65 (61 68)	**1,** Protein 7.7 (2.7 14.6)<br>Solution 92.3 (85.4 97.3)<br>**2,** Protein 37.5 (34.6 40.9)<br>Solution 62.5 (59.1 65.4)

Table S4. The resolved structural components before and after the interaction of Bax with SLBs composed of POPC containing Bcl-2 protein.

*Values in parentheses represent the 65% confidence intervals determined from MCMC resampling of the experimental data fits.

		Average Lipid Area per Molecule / Å²	Tails Thickness / Å	Tails Composition %	Head Group Thickness/ Å	Outer Head-group Composition %	Membrane Surface Bax/Lipid Complex Thickness / Å	Bax Surface Layer Composition %
**I) h-POPC: Bcl-2**		72.4 (68.4 76.9)	25.7 (24.3 27.3)	Lipid 79.8.8 (73.2 86.8)<br>Bcl-2 protein 9.9 (5.2 14.4)<br>Solution 10.2 (6.0 14.1)	10.2 (9.2 11.2)	Lipid 35.5 (32.6 39.7)<br>Bcl-2 protein 9.9 (5.2 14.4)<br>Solution 54.0 (48.7 59.2)		
	**+ h-Bax**	67.8 (63.7 72.5)	27.5 (25.7 29.3)	Lipid 76.7 (71.8 81.7)<br>Bcl-2 protein 8.3 (4.3 12.4)<br>Bax protein 10.4 (4.5 16.1)<br>Solution 2.3 (0.7 4.7)	12.9 (10.0 15.2)	Lipid 29.7 (25.6 37.2)<br>Bcl-2 protein 8.3 (4.3 12.4)<br>Bax protein 10.4 (4.5 16.1)<br>Solution 50.3 (48.2 55.0)	**1,** 88.4 (64.9 116.3)<br>**2,** 80.8 (58.6 94.3)<br>**Total:** 163 (142 192)	**1,** Protein 13.0 (10.3 15.8)<br>Solution 87.0 (84.2 89.7)<br>**2,** Protein 5.8 (3.7 7.9)<br>Solution 94.2 (92.1 96.3)
**Ii) h-POPC: Bcl-2**		73.0 (67.1 78.9)	25.5 (23.6 27.8)	Lipid 76.3 (69.6 83.6)<br>Bcl-2 protein 14.2 (9.3 19.2)<br>Solution 9.4 (4.3 14.0)	11.8 (10.7 12.9)	Lipid 29.3 (26.9 32.1)<br>Bcl-2 protein 14.2 (9.3 19.2)<br>Solution 56.5 (51.2 61.2)		
	**+ d-Bax**	63.7 (59.1 68.5)	29.3 (27.2 31.5)	Lipid 69.4 (63.6 75.3)<br>Bcl-2 protein 11.3 (7.0 15.7)<br>Bax protein 4.8 (2.9 6.7)<br>Solution 11.3 (7.3 15.5)	14.3 (12.0 16.2)	Lipid 26.2 (23.4 30.6)<br>Bcl-2 protein 11.3 (7.0 15.7)<br>Bax protein 4.8 (2.9 6.7)<br>Solution 57.0 (52.6 60.7)	**1,** 31.6 (14.0 50.7)<br>**2,** 37.9 (20.2 54.1)<br>**Total:** 68 (56 86)	**1,** Protein 9.0 (6.5 11.8)<br>Solution 91.0 (88.2 93.5)<br>**2,** Protein 6.9 (3.6 10.6)<br>Solution 93.1 (89.4 96.5)
**Iii) d-POPC: Bcl-2**		76.5 (69.1 84.2)	24.4 (22.2 27.0)	Lipid 48.6 (41.6 55.7)<br>Bcl-2 protein 39.9 (36.6 43.3)<br>Solution 11.5 (9.4 13.6)	9.3 (7.6 10.9)	Lipid 22.5 (21.2 24.0)<br>Bcl-2 protein 39.9 (38.5 43.3)<br>Solution 37.6 (31.3 42.4)	-	-
	**+ h-Bax**	81.3 (73.0 89.6)	22.9 (20.8 25.6)	Lipid 46.0 (38.0 52.8)<br>Bcl-2 protein 37.3 (33.2 42.1)<br>Bax protein 9.4 (4.0 14.7)<br>Solution 4.5 (1.4 8.3)	8.8 (7.0 10.7)	Lipid 22.0 (18.1 27.7)<br>Bcl-2 protein 37.3 (33.2 42.1)<br>Bax protein 9.4 (4.0 14.7)<br>Solution 30.3 (25.8 33.4)	**1,** 55 (50.6 58.8)<br>**2,** 52.8 (24.5 76.9)<br>**Total:** 108 (74 134)	**1,** Protein 26.2 (23.2 29.1)<br>Solution 73.8 (70.9 76.8)<br>**2,** Protein 4.5 (2.4 9.2)<br>Solution 95.5 (90.8 97.6)

Table S5. The time-resolved structural components during the interaction of Bax with SLB composed of d-POPC containing Bcl-2 protein. Structural components on the data set before and after Bax interaction shown in Table S3 part iii). Data sets measured in D2O subphase contrast only and parameters fixed other than the volume fraction and thickness parameters for Bax.
*Values in parentheses represent the 65% confidence intervals determined from MCMC resampling of the experimental data fits, note that when a prior has a hard limit, such as the relative multipliers below having a limit of 1, then the distribution of the posterior used for confidence interval estimation can be non-gaussian and the best fit value can lie slightly outside the 65% C.I.

Time since Bax injection	K	K_d	VF bilayer/%	VF first layer /%	VF second layer /%	Thickness of Bax layer 1/Å	Thickness of Bax layer 2/Å
0	0	0	0	0	0	0	0
47-83	0.47 (0.37 0.80)	0.43 (0.29 0.71)	4.7 (3.7 8.0)	12.3 (9.6 20.9)	2.1 (1.7 3.6)	23.7 (20.2 39.1)	22.7 (15.3 37.4)
83-120	0.59 (0.45 0.84)	0.49 (0.36 0.75)	5.9 (4.4 8.4)	15.5 (11.7 22.1)	2.7 (2.0 3.8)	27.0 (24.6 41.2)	25.9 (18.8 39.4)
120-156	0.63 (0.48 0.84)	0.59 (0.44 0.80)	6.3 (4.8 8.3)	16.5(12.7 21.9)	2.8 (2.2 3.8)	32.5 (26.7 44.1)	31.2 (23.1 42.3)
156-193	0.55 (0.44 0.75)	0.99 (0.50 0.92)	5.4 (4.4 7.4)	14.3(11.6 19.6)	2.5 (2.0 3.4)	54.5 (24.4 50.4)	52.3 (26.4 48.3)
193-234	0.67 (0.56 0.84)	0.81 (0.59 0.90)	6.6 (5.5 8.4)	17.4(14.6 22.1)	3.0 (2.5 3.8)	44.4 (30.6 49.8)	42.5 (30.9 47.7)
234-271	0.70 (0.58 0.85)	0.85 (0.60 0.92)	6.9 (5.8 8.5)	18.2(15.2 22.3)	3.1(2.6 3.8)	47.1 (32.0 50.5)	45.1 (31.7 48.4)
271-307	0.78 (0.64 0.91)	0.80 (0.64 0.91)	7.7 (6.4 9.1)	20.3(16.7 23.8)	3.5(2.9 4.1)	43.8 (35.2 50.2)	41.9 (33.6 48.2)
307-343	0.79 (0.63 0.90)	0.85 (0.64 0.92)	7.8 (6.3 8.9)	20.6(16.6 23.4)	3.5(2.8 4.0)	46.6 (34.9 50.7)	44.6 (34.0 48.6)
343-380	0.82 (0.69 0.92)	0.88 (0.71 0.94)	8.1(6.8 8.9)	21.4(18.0 24.1)	3.7(3.1 4.1)	48.2 (37.8 51.7)	46.2 (37.6 49.5)
380-416	0.86(0.72 0.93)	0.87(0.70 0.93)	8.5(7.2 9.2)	22.4(18.8 24.4)	3.8(3.2 4.2)	47.7 (39.6 51.4)	45.7 (36.9 49.2)
416-453	0.84 (0.70 0.92)	0.94 (0.74 0.95)	8.3(6.9 9.3)	21.9(18.2 24.2)	3.8(3.1 4.1)	51.5 (38.3 52.3)	49.4 (39.0 50.2)
453-489	0.89 (0.74 0.94)	1.00 (0.78 0.96)	8.8(7.4 9.2)	23.2(19.4 24.7)	4.0(3.3 4.2)	54.8 (40.8 52.9)	52.5 (41.3 50.7)
489-526	0.93 (0.77 0.95)	1.00(0.79 0.96)	9.2(7.7 9.4)	24.2(20.1 25.0)	4.2(3.5 4.3)	54.8 (42.4 53.1)	52.5 (41.5 50.8)
526-563	0.92 (0.78 0.96)	1.00 (0.82 0.97)	9.1(7.8 9.5)	24.0(20.4 25.0)	4.1(3.5 4.3)	55.0 (43.0 53.4)	52.7 (43.2 51.2)

563-603	0.96 (0.80 0.96)	0.97 (0.80 0.96)	9.5(7.9 9.5)	25.1(20.9 25.2)	4.3(3.6 4.3)	53.2 (43.9 53.4)	51.0 (42.4 50.9)
603-641	1.00 (0.80 0.96)	1.00 (0.82 0.97)	9.9(7.9 9.6)	26.1(20.9 25.2)	4.5(3.6 4.3)	55.0 (44.0 53.1)	52.7 (43.2 51.2)
641-693	0.95 (0.83 0.97)	1.00 (0.86 0.98)	9.4(8.3 9.6)	24.8(21.9 25.4)	4.3(3.8 4.4)	55.0 (46.0 53.4)	52.7 (45.6 51.6)
693-738	0.97 (0.84 0.97)	1.00 (0.86 0.98)	9.6(8.3 9.7)	25.3(22.0 25.4)	4.4(3.8 4.4)	55.0 (46.2 53.9)	52.7 (45.2 51.6)
Final structure	1	1	10.0%	26.2	4.5%	55.1 Å	52.8 Å

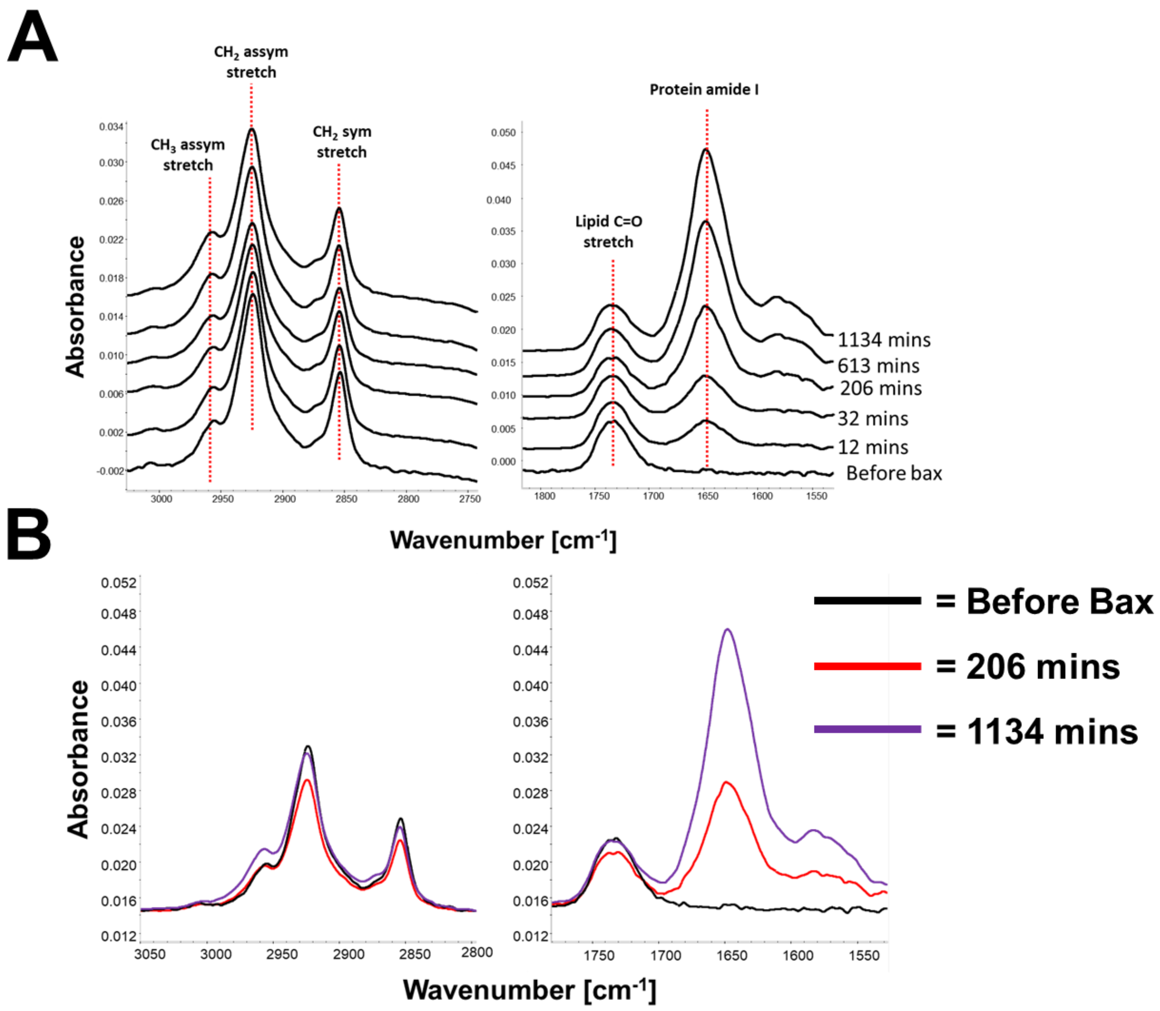

Figure S7. ATR-FTIR data obtained during the interaction of h-Bax with a h-POPC SLB. Changes in the CH stretch (A, Left) and Amide I (A, right) regions of the spectra are shown against time. An overlaid comparison of these regions is given; (B) showing the accumulation of Bax at the SLB coated surface and concurrent changes in the CH2 stretches.

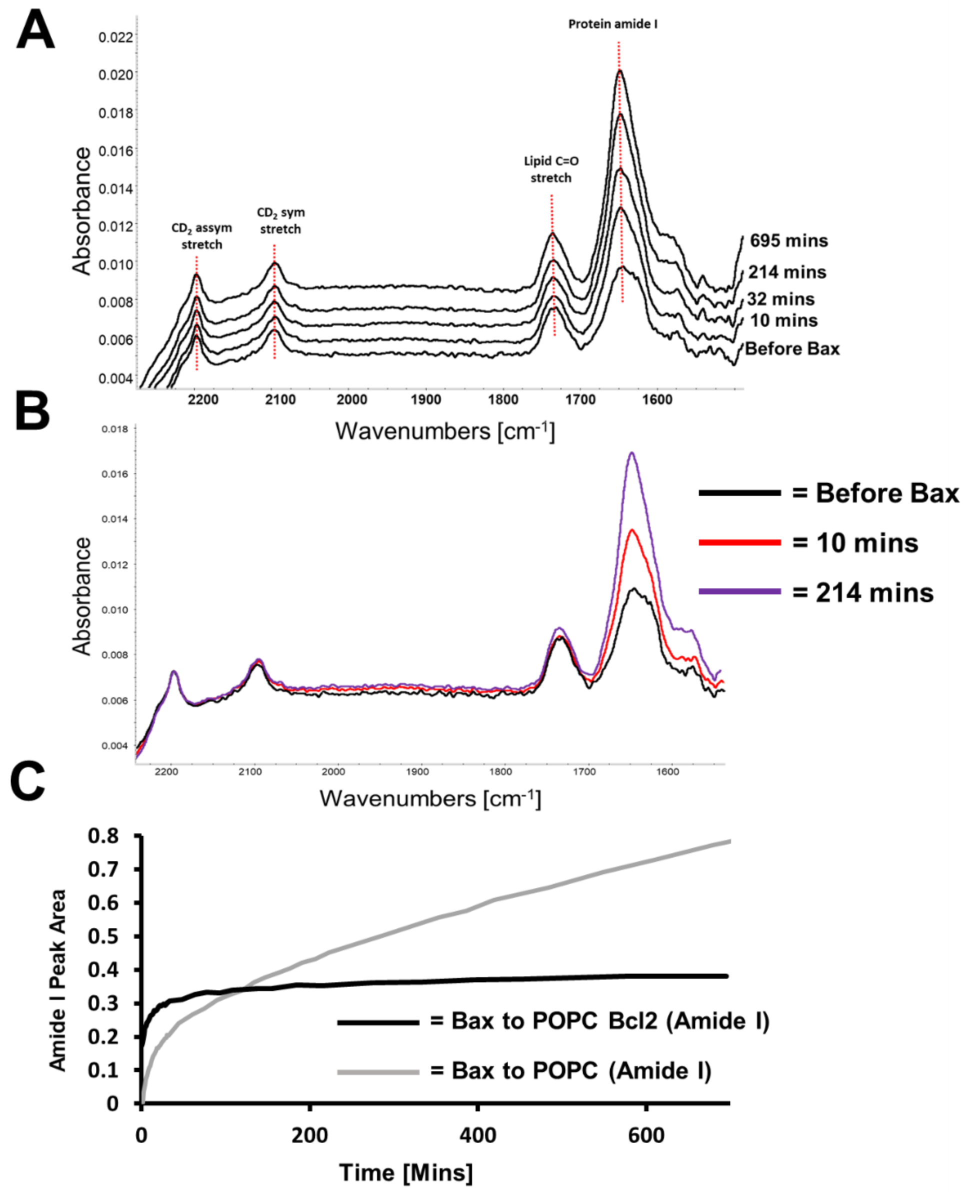

Figure S8. ATR-FTIR data from the interaction of h-Bax with a Bcl-2/d-POPC SLB. Changes in the CD stretching (lipid tail CD2 and CD3) and Amide I (Bcl-2 and Bax protein) region of the spectra against time are shown (A). An overlaid comparison of the spectra at differing times during the Bax binding process showing an increase in the amide I peak intensity no significant changes in the CD stretching region concurrent with Bax binding to, rather than disruption of the Bcl-2/POPC SLB (B). A comparison between the increase in the Amide I (protein carbonyl) band due to Bax binding to the POPC online (C, back line) and Bcl-2/POPC (C, grey line) SLBs, indicating that Bax binding to a Bcl-2 containing surface occurs on a faster time scale than Bax induced pore formation.

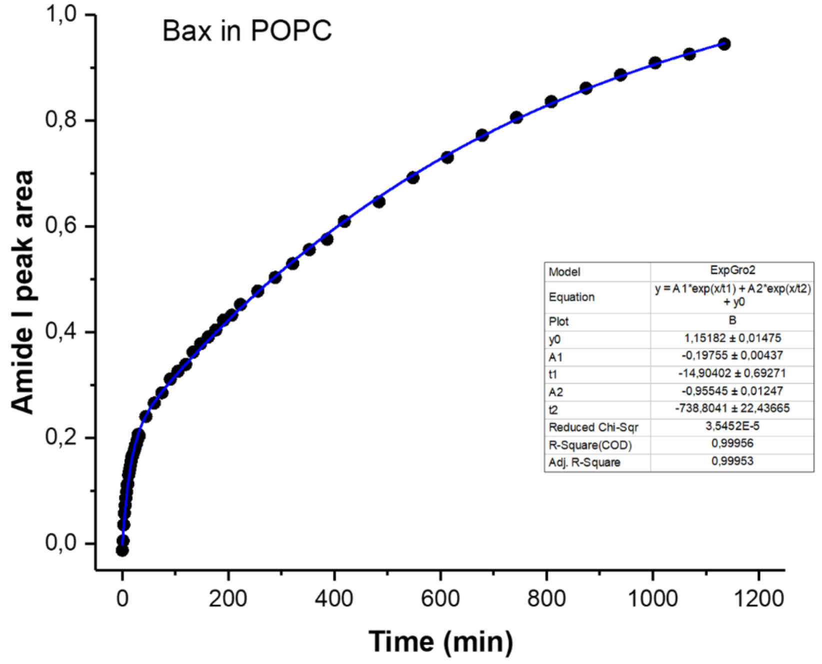

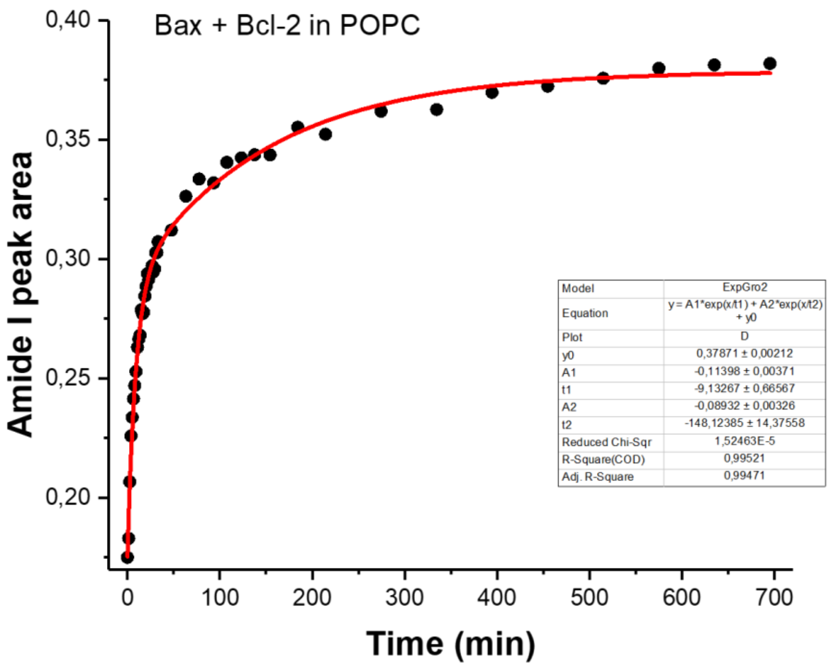

Figure S9. ATR-FTIR data showing the increase in Amide 1 peak area after addition of Bax to a SSB of POPC (top) and a SSB of POPC containing Bcl-2 (bottom) respectively. Collection times for each ATR-FTIR dataset were ~80s. Fits to exponential models show that association of Bax to these bilayers is a two-step process. The Bax association time constants for POPC bilayers were t1=-14.9 min and t2=738.8 min, and for POPC-Bcl-2 bilayer t1=-9.1 min and t2=148.1 min.

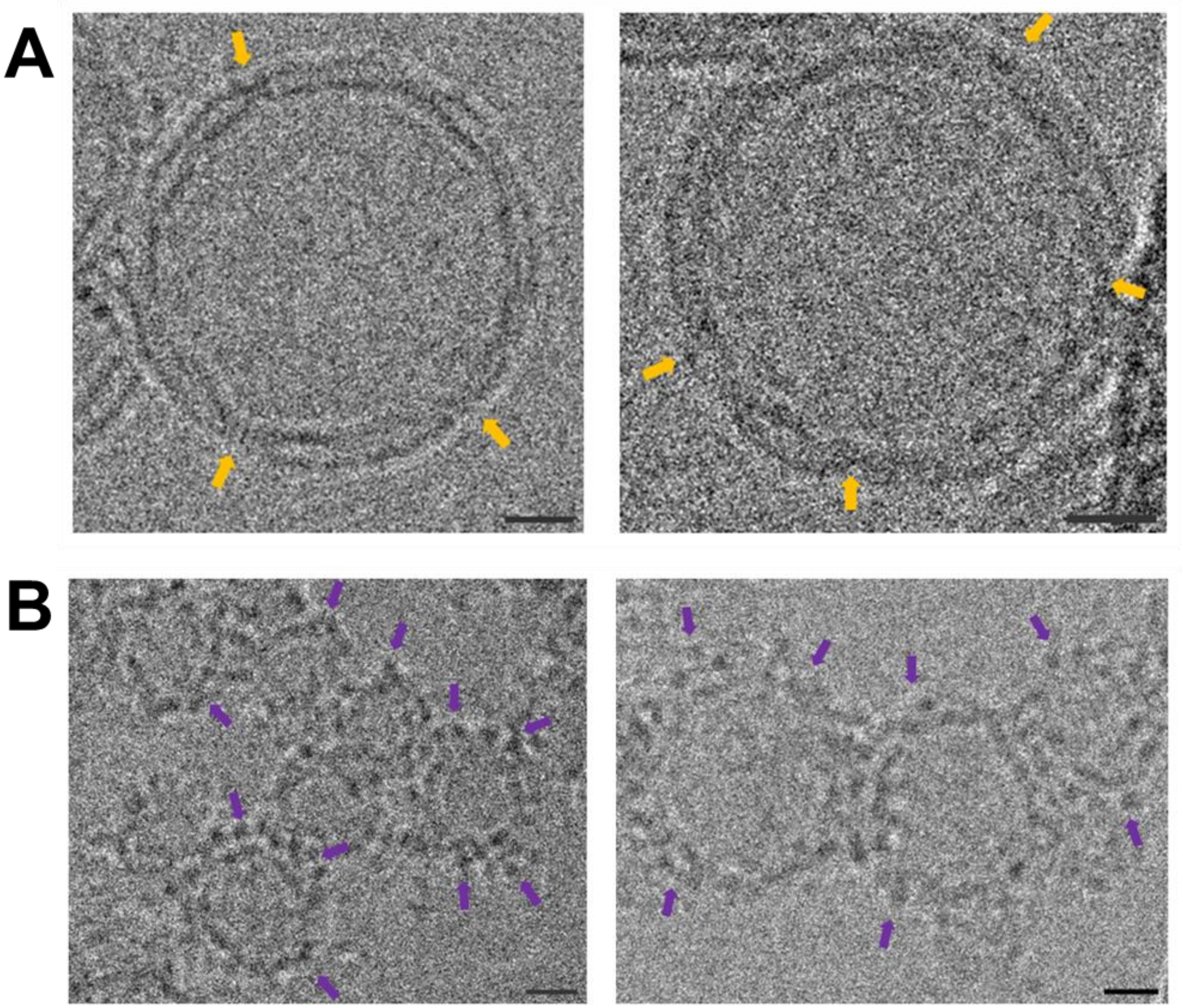

Figure S10. Selected EM microscopy image regions showing the presence of Bcl-2 (A, yellow arrows) and Bax (B, purple arrows) within and on the surface of h-Bcl-2 : d-POPC vesicles respectively. Images show d-POPC : h-Bcl-2 without (A) and after (B) the incubation in the presence of h-Bax for 60 minutes. The scale bar at the bottom right of each panel is 10 nm.

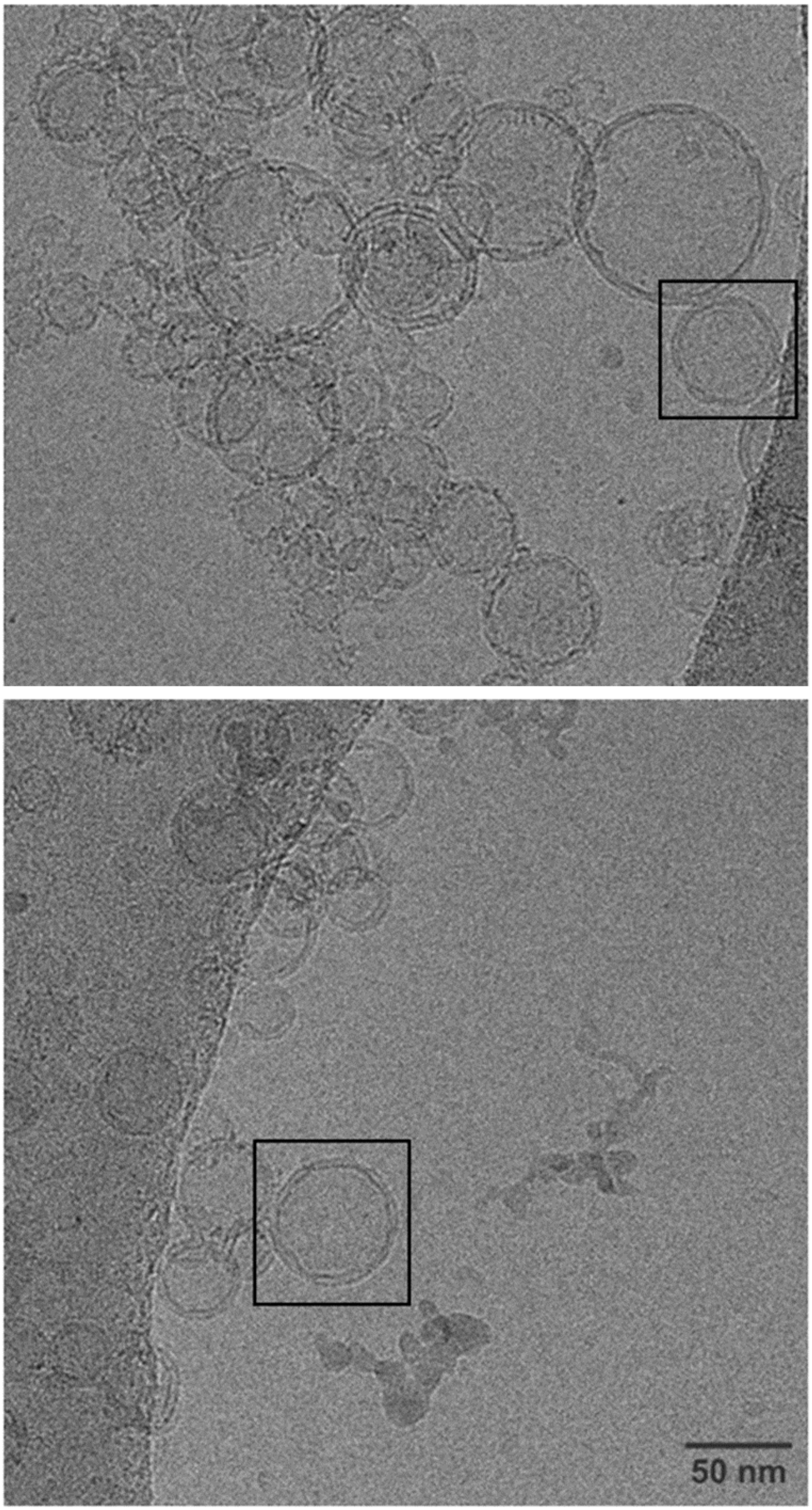

Figure S11. EM micrographs of d-POPC : h-Bcl-2 vesicles. Areas shown in black squares are shown expanded in Fig 1 and SI Fig 10.

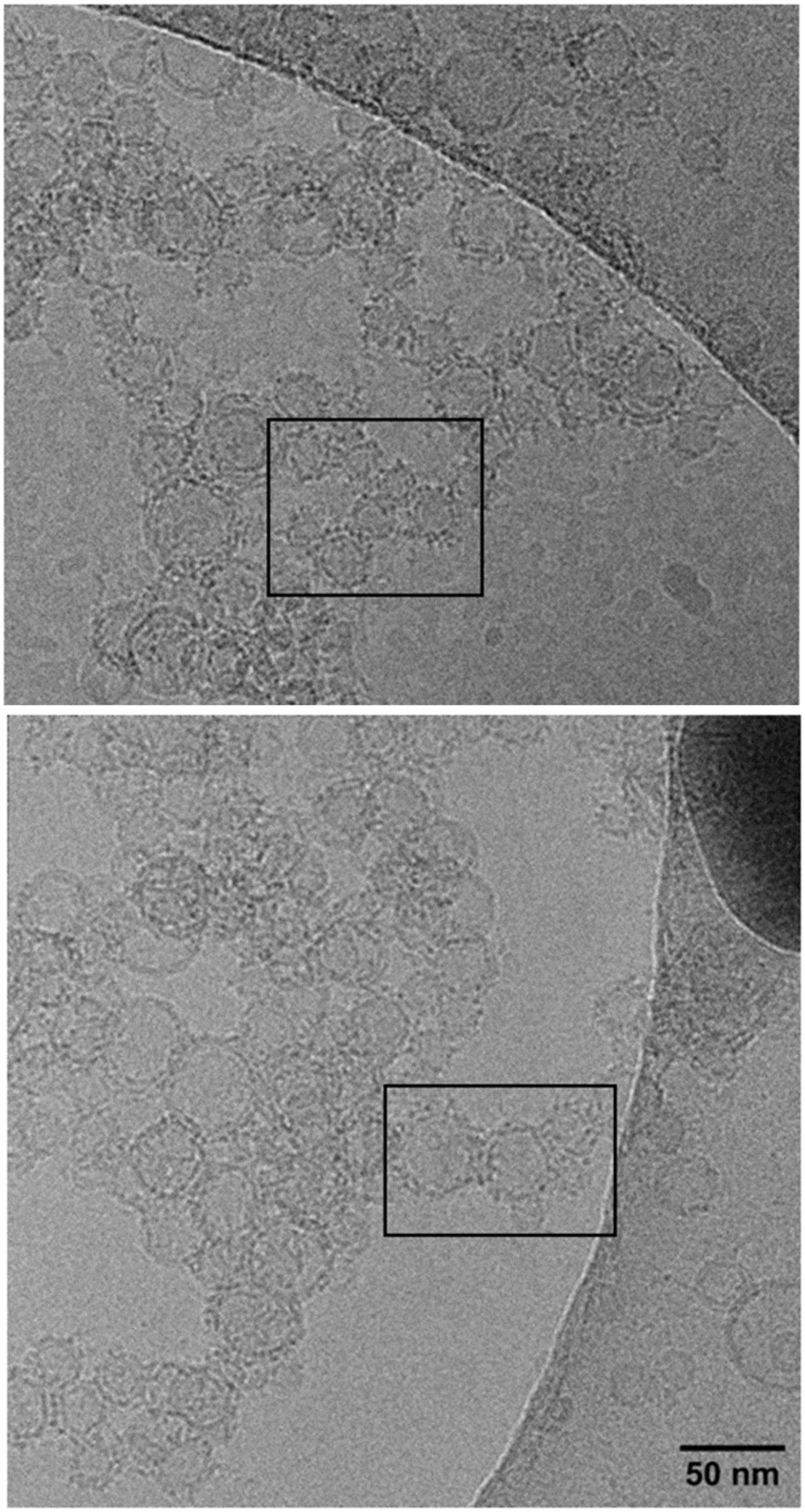

Figure S12. EM micrographs of h-Bax bound d-POPC : h-Bcl-2 vesicles. Areas shown in black squares are shown expanded in Fig 1 and SI Fig 10.

Section 3: Additional Modelling

Modelling of Bax structures for comparison with NR resolved Membrane Surface Bax distributions

Potential Bax dimer configurations based on the solution structure (1F16[12]) were generated using AlphaFold2-multimer *(*6), making use of ColabFold v1.5.5 [20]. The input protein sequence for Bax was obtained from UniProtKB assession number Q07812. Alphafold was set to detect potential templates [21] in the pdb100 database [22]) and perform AMBER relaxation [23] on the top 5 ranked structures. The radius (and subsequent diameter) of gyration was evaluated for each of the 5 relaxed structures. Sequence coverage was 100% for all positions. The predicted local distance difference test (pLDDT) scores and predicted aligned error (PAE) are shown in figure S14. As might be expected, the N-terminal residues show some of the highest uncertainty in position. Additionally, the recently described Bax polygon subunit of ring formation (9IXU[24]) was used to generate radii and diameters of gyration for activated Bax monomers, dimers and tetramers using the Crysol software[25]. Figure S13 shows a comparison of the structures and length scales of soluble Bax dimers (S13 A) and activated Bax dimers and tetramers (S13 B).

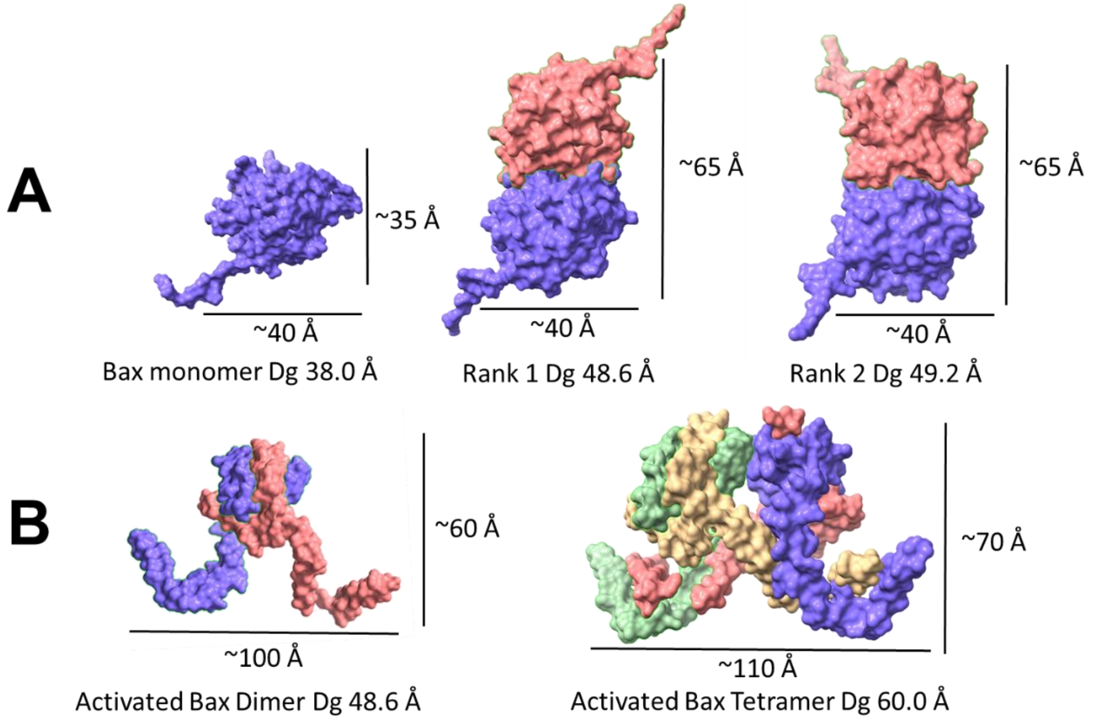

Figure S13. Bax monomer structure (PDB 1F16) and top two ranked dimer models with relative structural length scales (A) and Bax polygon subunit (PDB 9IXU) derived dimer and tetramer structures (B). Differing colours represent the different Bax subunits in the oligomer assembles.

For the soluble Bax structure (PDB 1F16), the radii and diameters of gyration obtained from the top ranked dimer models are highly comparable to the distances of the Bax distributions indicated from the reflectometry analysis, where the individual Bax distributions were found to be 52 – 68 Å in length (see main manuscript Table 2). Figure S13 (A) shows a comparison of a Bax monomer with the top 2 ranked dimer structures from AlphaFold-multimer modelling. In all cases the dimers showed a prolate structure with a minor axis of ~40 Å and a major axis of ~65 Å (not including the N-terminal tail regions).

Activated Bax dimers and tetramers derived from the activated the Bax polygon structure (PDB 9IXU[24]) show larger length scales (Fig 13 B) were also consistent with some of the Bax surface length scales resolved in the presence of Bcl-2 (manuscript table 2).

Table S6. Radius and diameter of gyration of top 5 relaxed Bax dimer structures from AlphaFold modelled from the Bax solution structure (PDB entry 1F16).

Structure Rank	Radius of gyration (Å)	Diameter of gyration (Å)
Monomer structure pdb 1F16	19.0	38.0
Dimer Rank 1	24.3	48.6
Dimer Rank 2	24.6	49.2
Dimer Rank 3	24.9	49.8
Dimer Rank 4	24.5	49.0
Dimer Rank 5	24.7	49.4

Table S7. Radius and diameter of gyration of activated Bax monomer, dimer and tetramers from the activated Bax polygon subunit (PDB 9IXU).

Structure	Radius of gyration (Å)	Diameter of gyration (Å)
Monomer (Chain A)	24.4	48.8
Monomer (Chain B)	25.2	50.4
Dimer (AB)	27.1	54.2
Tetramer (ABCD)	30.0	60.0

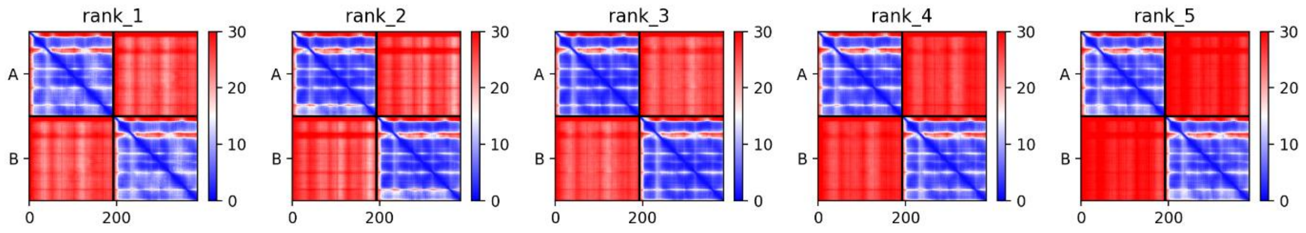

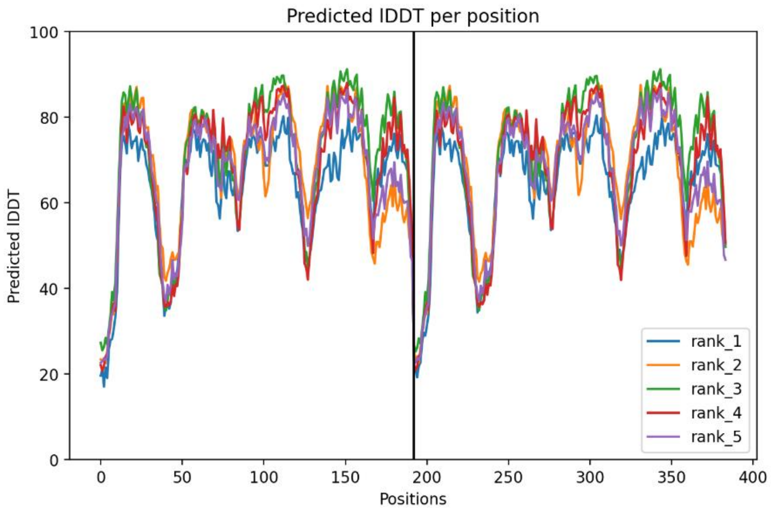

Figure S14. Predicted aligned error and pLDDT scores for AlphaFold prediction

Section 4: Production of functional, full-length Bcl-2 and Bax proteins

Preparation of protonated (h-Bcl-2) and deuterated (d-Bcl-2) protein:

The production and purification of pure, fully functional human Bcl-2 fully protonated (h-forms) or deuterated (d-forms) were developed by us previously to ensure sufficient mg quantities of material for biophysical studies. Åden et al.[26] describes the protocol used for production and refolding of h-Bcl-2 including functionality assays and purity assesments. Reconstitution of detergent solubilized Bcl-2 into bilayers is described in detail by Wallgren et al.[27] and the production of d-Bcl-2 is described in detail by Mustaq et al[28].

In short, Bcl-2 was expressed and purified following the method previously described by Åden et al.[26] and deuterated to >90%. Transformed BL21(DE3) Rosetta™ cells with Bcl-2 encoded in a pET-15b vector (Novagen) were cultured in media with step-wise increases in deuterium oxide concentration with M9 media with increasing D_2O content (with 50% being the initial D_2O content). Cell pellets from each round was collected to transferred to Media with higher D_2O content. The final deuteration content of the media was 99.8% D_2O. Deuterated Bcl-2 was harvested from this culture, purified and reconstituted as described previously[26-28].

Preparation of protonated (h-Bax) and deuterated (d-Bax) protein:

Bax production was similar to that described for Bcl-2 except Bax was solution soluble and Bcl-2 was not. In short, mg quantity production of soluble fully functional, human full length Bax was established recently by us[29]. The production of d-Bax is described in detail by Clifton et al[5] using a similar strategy as for Bcl-2 in adapting cells to increasing D_2O concentrations. Deuteration (>90%) and functionality were assessed using methods described previously[5,29,30].

Bax activation state:

Purified Bax was in an active monomeric state as suggested by biophysical interaction studies by us[29] and others[7,9,10,32,33]. It is suggested that this activation of Bax happens during purification, when the protein is in contact with fatty acids during ultrasonication of the cell pellet; as mentioned by Yethon *et al*[33].

References

(1) Hughes, A. V. *RasCal 2019*. https://github.com/arwelHughes/RasCAL_2019.
(2) Tristram-Nagle, S.; Liu, Y.; Legleiter, J.; Nagle, J. F. Structure of Gel Phase DMPC Determined by X-Ray Diffraction. *Biophys. J.* **2002**, *83* (6), 3324–3335. https://doi.org/10.1016/S0006-3495(02)75333-2.
(3) Haario, H.; Laine, M.; Mira, A.; Saksman, E. DRAM: Efficient Adaptive MCMC. *Stat Comput* **2006**, *16* (4), 339–354. https://doi.org/10.1007/s11222-006-9438-0.
(4) Tatulian, S. A. Attenuated Total Reflection Fourier Transform Infrared Spectroscopy: A Method of Choice for Studying Membrane Proteins and Lipids. *Biochemistry* **2003**, *42* (41), 11898–11907. https://doi.org/10.1021/bi034235+.
(5) Clifton, L. A.; Wacklin-Knecht, H. P.; Ådén, J.; Mushtaq, A. U.; Sparrman, T.; Gröbner, G. Creation of Distinctive Bax-Lipid Complexes at Mitochondrial Membrane Surfaces Drives Pore Formation to Initiate Apoptosis. *Sci. Adv.* **2023**, *9* (22). https://doi.org/10.1126/sciadv.adg7940.
(6) Shamas-Din, A.; Bindner, S.; Chi, X.; Leber, B.; Andrews, D. W.; Fradin, C. Distinct Lipid Effects on TBid and Bim Activation of Membrane Permeabilization by Pro-Apoptotic Bax. *Biochem. J.* **2015**, *467* (3), 495–505. https://doi.org/10.1042/BJ20141291.
(7) Mystek, P.; Singh, V.; Horváth, M.; Honzejková, K.; Riegerová, P.; Evci, H.; Hof, M.; Obšil, T.; Šachl, R. The Minimal Membrane Requirements for BAX-Induced Pore Opening upon Exposure to Oxidative Stress. *Biophys. J.* **2024**, *123* (20), 3519–3532. https://doi.org/10.1016/j.bpj.2024.08.017.
(8) Lai, Y.-C.; Li, C.-C.; Sung, T.-C.; Chang, C.-W.; Lan, Y.-J.; Chiang, Y.-W. The Role of Cardiolipin in Promoting the Membrane Pore-Forming Activity of BAX Oligomers. *Biochim. Biophys. Acta - Biomembr.* **2019**, *1861* (1), 268–280. https://doi.org/10.1016/j.bbamem.2018.06.014.
(9) Vasquez-Montes, V.; Rodnin, M. V.; Kyrychenko, A.; Ladokhin, A. S. Lipids Modulate the BH3-Independent Membrane Targeting and Activation of BAX and Bcl-XL. *Proc. Natl. Acad. Sci.* **2021**, *118* (37). https://doi.org/10.1073/pnas.2025834118.
(10) Shamas-Din, A.; Satsoura, D.; Khan, O.; Zhu, W.; Leber, B.; Fradin, C.; Andrews, D. W. Multiple Partners Can Kiss-and-Run: Bax Transfers between Multiple Membranes and Permeabilizes Those Primed by TBid. *Cell Death Dis.* **2014**, *5* (6), e1277–e1277. https://doi.org/10.1038/cddis.2014.234.
(11) Czabotar, P. E.; Garcia-Saez, A. J. Mechanisms of BCL-2 Family Proteins in Mitochondrial Apoptosis. *Nat. Rev. Mol. Cell Biol.* **2023**, *24* (10), 732–748. https://doi.org/10.1038/s41580-023-00629-4.
(12) Suzuki, M.; Youle, R. J.; Tjandra, N. Structure of Bax. *Cell* **2000**, *103* (4), 645–654. https://doi.org/10.1016/S0092-8674(00)00167-7.
(13) Gahl, R. F.; He, Y.; Yu, S.; Tjandra, N. Conformational Rearrangements in the Pro-Apoptotic Protein, Bax, as It Inserts into Mitochondria. *J. Biol. Chem.* **2014**, *289* (47), 32871–32882. https://doi.org/10.1074/jbc.M114.593897.
(14) Lv, F.; Qi, F.; Zhang, Z.; Wen, M.; Kale, J.; Piai, A.; Du, L.; Wang, S.; Zhou, L.; Yang, Y.; Wu, B.; Liu, Z.; del Rosario, J.; Pogmore, J.; Chou, J. J.; Andrews, D. W.; Lin, J.; OuYang, B. An Amphipathic Bax Core Dimer Forms Part of the Apoptotic Pore Wall in the Mitochondrial ⎵ membrane. *EMBO J.* **2021**, *40* (14). https://doi.org/10.15252/embj.2020106438.
(15) Hauseman, Z. J.; Harvey, E. P.; Newman, C. E.; Wales, T. E.; Bucci, J. C.; Mintseris, J.; Schweppe, D. K.; David, L.; Fan, L.; Cohen, D. T.; Herce, H. D.; Mourtada, R.; Ben-Nun, Y.; Bloch, N. B.; Hansen, S. B.; Wu, H.; Gygi, S. P.; Engen, J. R.; Walensky, L. D. Homogeneous Oligomers of Pro-Apoptotic BAX Reveal Structural Determinants of Mitochondrial Membrane Permeabilization. *Mol. Cell* **2020**, *79* (1), 68-83.e7. https://doi.org/10.1016/j.molcel.2020.05.029.
(16) Ader, N. R.; Hoffmann, P. C.; Ganeva, I.; Borgeaud, A. C.; Wang, C.; Youle, R. J.; Kukulski, W. Molecular and Topological Reorganizations in Mitochondrial Architecture Interplay during Bax-Mediated Steps of Apoptosis. *Elife* **2019**, *8*. https://doi.org/10.7554/eLife.40712.
(17) Schweighofer, S. V.; Jans, D. C.; Keller-Findeisen, J.; Folmeg, A.; Ilgen, P.; Bates, M.; Jakobs, S. Endogenous BAX and BAK Form Mosaic Rings of Variable Size and Composition on Apoptotic Mitochondria. *Cell Death Differ.* **2024**, *31* (4), 469–478. https://doi.org/10.1038/s41418-024-01273-x.
(18) Myatt, D.; Clifton, L. A. *Biomolecular Scattering Length Density Calculator*. http://psldc.isis.rl.ac.uk/Psldc/.
(19) Evans, R.; O'Neill, M.; Pritzel, A.; Antropova, N.; Senior, A.; Green, T.; Žídek, A.; Bates, R.; Blackwell, S.; Yim, J.; Ronneberger, O.; Bodenstein, S.; Zielinski, M.; Bridgland, A.; Potapenko, A.; Cowie, A.; Tunyasuvunakool, K.; Jain, R.; Clancy, E.; Kohli, P.; Jumper, J.; Hassabis, D. Protein Complex Prediction with AlphaFold-Multimer. *bioRxiv* **2021**. https://doi.org/10.1101/2021.10.04.463034.
(20) Mirdita, M.; Schütze, K.; Moriwaki, Y.; Heo, L.; Ovchinnikov, S.; Steinegger, M. ColabFold: Making Protein Folding Accessible to All. *Nat. Methods* **2022**, *19* (6), 679–682. https://doi.org/10.1038/s41592-022-01488-1.
(21) Mirdita, M.; Steinegger, M.; Söding, J. MMseqs2 Desktop and Local Web Server App for Fast, Interactive Sequence Searches. *Bioinformatics* **2019**, *35* (16), 2856–2858.

https://doi.org/10.1093/bioinformatics/bty1057.
(22) van Kempen, M.; Kim, S. S.; Tumescheit, C.; Mirdita, M.; Lee, J.; Gilchrist, C. L. M.; Söding, J.; Steinegger, M. Fast and Accurate Protein Structure Search with Foldseek. *Nat. Biotechnol.* **2024**, *42* (2), 243–246. https://doi.org/10.1038/s41587-023-01773-0.
(23) Eastman, P.; Swails, J.; Chodera, J. D.; McGibbon, R. T.; Zhao, Y.; Beauchamp, K. A.; Wang, L.-P.; Simmonett, A. C.; Harrigan, M. P.; Stern, C. D.; Wiewiora, R. P.; Brooks, B. R.; Pande, V. S. OpenMM 7: Rapid Development of High Performance Algorithms for Molecular Dynamics. *PLOS Comput. Biol.* **2017**, *13* (7), e1005659. https://doi.org/10.1371/journal.pcbi.1005659.
(24) Zhang, Y.; Tian, L.; Huang, G.; Ge, X.; Kong, F.; Wang, P.; Xu, Y.; Shi, Y. Structural Basis of BAX Pore Formation. *Science (80-.).* **2025**, *388* (6754). https://doi.org/10.1126/science.adv4314.
(25) Svergun, D.; Barberato, C.; Koch, M. H. J. CRYSOL – a Program to Evaluate X-Ray Solution Scattering of Biological Macromolecules from Atomic Coordinates. *J. Appl. Crystallogr.* **1995**, *28* (6), 768–773. https://doi.org/10.1107/S0021889895007047.
